# SARS-CoV-2 envelope protein impairs airway epithelial barrier function and exacerbates airway inflammation via increased intracellular Cl^−^ concentration

**DOI:** 10.1038/s41392-024-01753-z

**Published:** 2024-03-25

**Authors:** Jian-Bang Xu, Wei-Jie Guan, Yi-Lin Zhang, Zhuo-Er Qiu, Lei Chen, Xiao-Chun Hou, Junqing Yue, Yu-Yun Zhou, Jie Sheng, Lei Zhao, Yun-Xin Zhu, Jing Sun, Jincun Zhao, Wen-Liang Zhou, Nan-Shan Zhong

**Affiliations:** 1 State Key Laboratory of Respiratory Disease, National Clinical Research Center for Respiratory Disease, National Center for Respiratory Medicine, Department of Respiratory and Critical Care Medicine, Guangzhou Institute of Respiratory Health, The First Affiliated Hospital of Guangzhou Medical University, Guangzhou, P. R. China; 2 Department of Thoracic Surgery, Guangzhou Institute for Respiratory Health, The First Affiliated Hospital of Guangzhou Medical University, Guangzhou, P. R. China; 3 Guangzhou National Laboratory, Guangzhou, P. R. China; 4 School of Life Sciences, Sun Yat-sen University, Guangzhou, P. R. China; 5 Guangdong Provincial Key Laboratory of Pharmaceutical Functional Genes, School of Life Sciences, Sun Yat-sen University, Guangzhou, P. R. China; 6 Department of Physiology, School of Basic Medical Sciences, Guangzhou Medical University, Guangzhou, P. R. China

**Keywords:** Inflammation, Innate immunity

## Abstract

Severe acute respiratory syndrome coronavirus 2 (SARS-CoV-2) infection disrupts the epithelial barrier and triggers airway inflammation. The envelope (E) protein, a core virulence structural component of coronaviruses, may play a role in this process. Pathogens could interfere with transepithelial Cl^−^ transport via impairment of the cystic fibrosis transmembrane conductance regulator (CFTR), which modulates nuclear factor κB (NF-κB) signaling. However, the pathological effects of SARS-CoV-2 E protein on airway epithelial barrier function, Cl^−^ transport and the robust inflammatory response remain to be elucidated. Here, we have demonstrated that E protein down-regulated the expression of tight junctional proteins, leading to the disruption of the airway epithelial barrier. In addition, E protein triggered the activation of Toll-like receptor (TLR) 2/4 and downstream c-Jun N-terminal kinase (JNK) signaling, resulting in an increased intracellular Cl^−^ concentration ([Cl^−^]_i_) via up-regulating phosphodiesterase 4D (PDE4D) expression in airway epithelial cells. This elevated [Cl^−^]_i_ contributed to the heightened airway inflammation through promoting the phosphorylation of serum/glucocorticoid regulated kinase 1 (SGK1). Moreover, blockade of SGK1 or PDE4 alleviated the robust inflammatory response induced by E protein. Overall, these findings provide novel insights into the pathogenic role of SARS-CoV-2 E protein in airway epithelial damage and the ongoing airway inflammation during SARS-CoV-2 infection.

## Introduction

Severe respiratory syndrome coronavirus 2 (SARS-CoV-2), the pathogen responsible for the global coronavirus disease 2019 (COVID-19) pandemic, has posed significant challenges to public health worldwide.^1,2^ SARS-CoV-2 could disrupt the alveolar epithelial barrier and elicit heightened airway inflammation, contributing to the labored breathing and, in severe cases, progression to acute respiratory failure.^3,4^ However, the precise molecular mechanisms underlying the disrupted epithelial barrier function and augmented inflammation is not entirely clear. Understanding the pathogenic mechanisms of SARS-CoV-2 could aid in the identification of novel targets for clinical intervention. SARS-CoV-2 is an enveloped virus with a positive-sense single-stranded RNA genome. The virus could disrupt the epithelial integrity and exacerbate the inflammation in the airway epithelia, the first-line defense against viral infection.^5^ SARS-CoV-2 encodes four structural proteins - the Spike (S), Envelope (E), Membrane (M) and Nucleocapsid (N) proteins.^6^ The S protein mediates viral binding to the host receptor angiotensin-converting enzyme 2 (ACE2) and virus–cell membrane fusion. The M protein plays a vital role in viral assembly, while the N protein is essential for viral genome packaging and survival. Notably, the E protein serves as a core structural virulence factor that plays a crucial role in maintaining the viral life cycle (such as viral assembly and budding).^7,8^ Several studies have explored the pathogenic roles of the SARS-CoV-2 S, M and N proteins.^9–13^ However, little is known regarding the effects and mechanisms of the SARS-CoV-2 E protein on epithelial tight junctions and mucosal inflammation.

The airway barrier consists of tight junctions, mucociliary clearance and immune cells. The tight junctions are supramolecular entities composed of claudin family transmembrane proteins, which linked to the actin cytoskeleton via cytosolic PDZ domain containing scaffold proteins. Tight junctions regulate the paracellular permeability of molecule and modulate the innate immunity by establishing a physical barrier through maintenance of the integrity and cell polarity of the mucosal monolayer.^14^ However, during respiratory virus infection, the barrier function could be impaired due to the dysregulated expression of tight junction proteins, leading to the passage of invading pathogens into the subepithelial space.^14^ Previous studies have demonstrated that high viral loads of SARS-CoV-2 could disrupt airway epithelial tight junctions,^15,16^ partly involving the interactions with Proteins Associated with Lin Seven 1 (PALS1).^17,18^ Additionally, SARS-CoV-2 E protein might also interact with tight junction protein zona occluden-1 (ZO-1), leading to epithelial tight junction damage.^19^ These observations indicated the critical role of the SARS-CoV-2 E protein in triggering epithelial barrier dysfunction.

Mucociliary clearance plays a crucial role in maintaining homeostasis of the airway luminal microenvironment, ensuring efficient clearance of the invading pathogens. The effectiveness of mucociliary clearance is partially dependent on the dynamics of water and ion transportation. Chloride anion (Cl^−^) is the principal anion in the human body and the intracellular Cl^−^ concentration ([Cl^−^]_i_) is tightly regulated by multiple Cl^−^ channels and transporters.^20^ Recent evidence suggests that intracellular Cl^−^ may modulate signaling, and that the disruption of intracellular Cl^−^ homeostasis may be associated with alterations in diverse cellular functions, such as inflammation and immune disorders.^11,21,22^ Consequently, the accumulation of intracellular Cl^−^ may play a fundamental role in amplifying ongoing airway inflammation following pathogen (e.g., viruses) infections. Cystic fibrosis transmembrane conductance regulator (CFTR) is a 3’,5’-cyclic monophosphate (cAMP) activated Cl^−^ channel expressed in the apical membrane of epithelial cells throughout the body. In epithelia, the infection of various pathogens may cause decreased expression or dysfunction of CFTR, leading to the impaired anion secretion and disequilibrium of intracellular Cl^−^.^23,24^ Intriguingly, Cl^−^ efflux which is mediated by CFTR and TMEM16 has been implicated in SARS-CoV-2 entry and replication,^13,25^ indicating a role of intracellular Cl^−^ signaling during SARS-CoV-2 infection.

Toll-like receptors (TLRs) are pattern recognition receptors (PRRs) which initiate the inflammatory responses and innate defense responses during infection through recognizing pathogen-associated molecular patterns (PAMPs). TLR2 was proven to recognize S protein and E protein of SARS-CoV-2, playing a crucial role in the development of COVID-19.^9,26^ Mitogen activated protein kinase (MAPK) cascades are highly conserved signaling molecules responsible for transduction of extracellular stimuli into cellular signals. Three major MAPK pathways in mammalian cells have been clearly identified, including extracellular signal-regulated kinase (ERK, also known as p42/p44 MAPK), c-Jun N-terminal kinase (JNK, also known as stress-activated protein kinase, SAPK) and p38 kinase.^27^ The MAPK pathway has been shown to be activated by various viruses, thus representing the critical targets for mitigating the virus-induced inflammatory cytokine storm.^28^ Therefore, investigating the involvement of TLR signaling and MAPK pathway during SARS-CoV-2 infection may help to elucidate the immune pathogenesis of COVID-19 and explore valuable therapeutic targets for antiviral therapy. In this study, therefore, we aimed to investigate the pathological effects and the underlying mechanisms of SARS-CoV-2 E protein on airway epithelial integrity and inflammatory responses.

## Results

### SARS-CoV-2 E protein impairs airway epithelial integrity

**Fig. 1:**
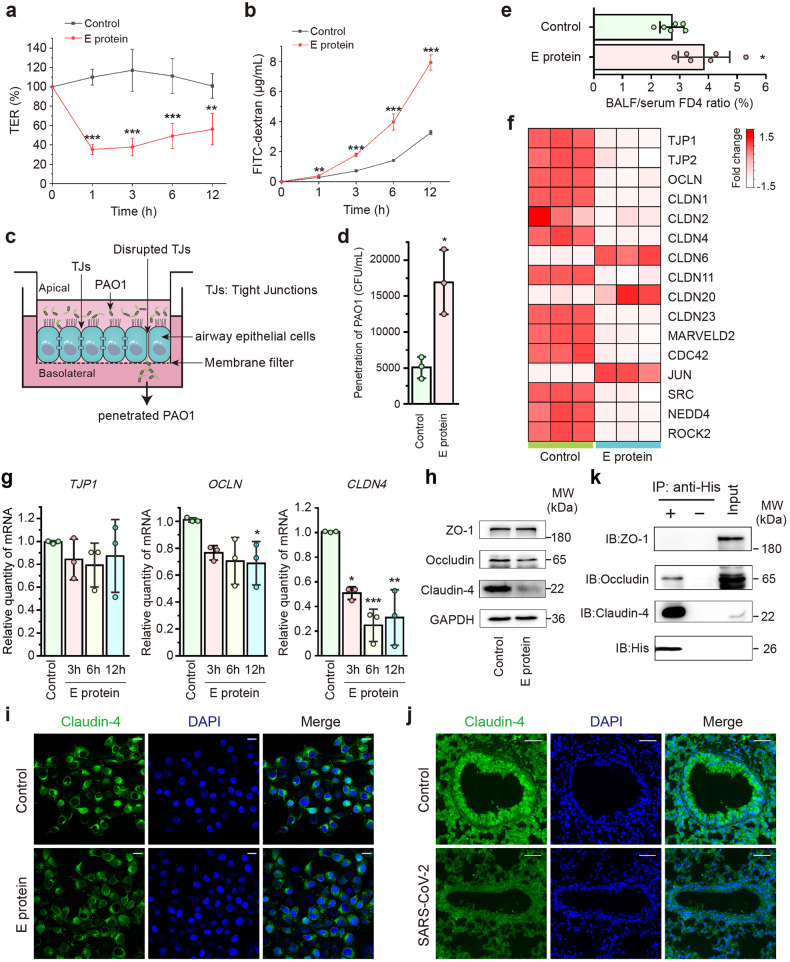
SARS-CoV-2 envelope (E) protein disrupted barrier function and altered the expression of tight junction proteins in airway epithelial cells. **a**, **b** 16HBE14o- monolayers were stimulated with E protein (50 μg/ml) apically for the indicated time points. **a** Transepithelial resistance (TER) values were measured and shown relative to the TER before E protein stimulation (*n* = 5). **b** Fluorescence intensity of fluorescein isothiocyanate-dextran (4 kDa, FD4) flux across cell monolayers were monitored (*n* = 3). **c** Diagram of *Pseudomonas aeruginosa* strain PAO1 penetration through airway epithelial cell monolayer. Airway epithelial cells were seeded into the upper chamber of plate inserts with a 3 μm pore size membrane, which allowed the passage of PAO1. Cell monolayer with tight junctions (TJs) were infected apically with PAO1 and the penetrated bacteria from the basolateral medium in different groups were enumerated. The figure was created using Adobe Illustrator. **d** The colony-forming units (CFU) of penetrated PAO1 obtained from the plate counting technique. 16HBE14o- monolayers were infected with PAO1 at a multiplicity of infection (MOI) of 20 for 2 h, with or without E protein (50 μg/ml) stimulation (*n* = 3). **e** ICR mice were injected with FD4 after the stimulation of E protein (300 μg/ml) or saline for 6 h. The fluorescence ratio of BALF and serum was calculated (*n* = 6). **f** Heatmap of tight junction-related genes with significant changes in BEAS-2B cells stimulated with E protein (50 μg/ml) for 12 h. **g** Quantitative real-time PCR analyses showing the expression of ZO-1, occludin and claudin-4 in BEAS-2B cells stimulated with E protein (50 μg/ml) for the indicated time points (*n* = 3). Data are shown as means ± S.D. ^*^P < 0.05, ^**^P < 0.01, ^***^P < 0.001 versus the control group. **h** Representative immunoblots showing the expression level of ZO-1, occludin and claudin-4 in BEAS-2B cells after stimulation with E protein (50 μg/ml) for the indicated time points. GAPDH served as a loading control. **i** Immunofluorescence images showing the expression of claudin-4 in BEAS-2B cells, in the absence or presence of E protein (50 μg/ml) stimulation. Scale bar = 20 μm. **j** Immunofluorescence staining of lung sections showing the expression of claudin-4 in Ad5-hACE2 transgenic mice with or without SARS-CoV-2 infection (1 × 10^5^ PFU). Scale bar = 50 μm. **k** After incubation with E protein (50 μg/ml), the co-immunoprecipitates and the total lysates (Input) were analyzed by immunoblotting with the indicated antibodies

SARS-CoV-2 infection causes acute lung injury by impairing airway epithelial barrier function. We thus initially examined the effects of SARS-CoV-2 E protein on airway epithelial integrity. The confluent monolayers of human airway epithelial cells were apically treated with E protein at Day 9, when the monolayers with tight junctions were formed (Supplementary Fig. 1). E protein, but not the solvents per se, significantly decreased the transepithelial resistance (TER) (Fig. 1a and Supplementary Fig. 5a), which was parallel to a significant increase in the paracellular flux of fluorescein isothiocyanate-dextran with average molecular weight of 4 kDa (FD4) (Fig. 1b and Supplementary Fig. 5b). Next, we evaluated the penetration of *P. aeruginosa* strain PAO1 through the monolayer (Fig. 1c). After stimulation with E protein, the airway epithelial cells were apically infected with PAO1 for 3 h, and the penetration of PAO1 through the epithelial monolayer was markedly facilitated (Fig. 1d and Supplementary Fig. 2), while the epithelial cell viability was not significantly altered (Supplementary Fig. 3). These findings confirmed that SARS-CoV-2 E protein could impair airway epithelial barrier and enhance the susceptibility to other core respiratory pathogens. Similarly, the in vivo study with mice showed that E protein induced airway epithelial injury, as evidenced by an increased ratio of the bronchoalveolar lavage fluid (BALF) to serum for FD4 (Fig. 1e).

The integrity of airway epithelium is maintained by tight junction–associated proteins, such as ZO-1, occludin, and claudins,^29,30^ we therefore examined the expression patterns of the differentially expressed genes (DEGs) related to tight junctions in airway epithelial cells after E protein stimulation. Notably, E protein triggered significant alteration in transcriptional gene expression of various tight junction–associated proteins, including occludin and claudin 4 (Fig. 1f, g). Consistent with the mRNA expression levels, E protein markedly decreased the protein expression levels of occludin and claudin 4 (Fig. 1h, i and Supplementary Fig. 4a, b). These findings were further verified in a murine model of SARS-CoV-2 infection by transfection with hACE2, revealing similar expression patterns of these proteins (Fig. 1j and Supplementary Fig. 4c, d).

The interactions between SARS-CoV-2 E protein and host proteins, such as PALS1, have been implicated in the disruption of epithelial integrity.^18^ Here, we further revealed that His-tagged E protein could directly interact with the cardinal tight junction proteins, evidenced by the co-immunoprecipitation of anti-His antibody with both occludin and claudin-4 (Fig. 1k). Our findings have collectively indicated that SARS-CoV-2 E protein may interact with and regulate the expression levels of cardinal tight junction proteins, which elicited airway epithelial barrier dysfunction.

### SARS-CoV-2 E protein induces robust inflammation in airway epithelial cells

**Fig. 2:**
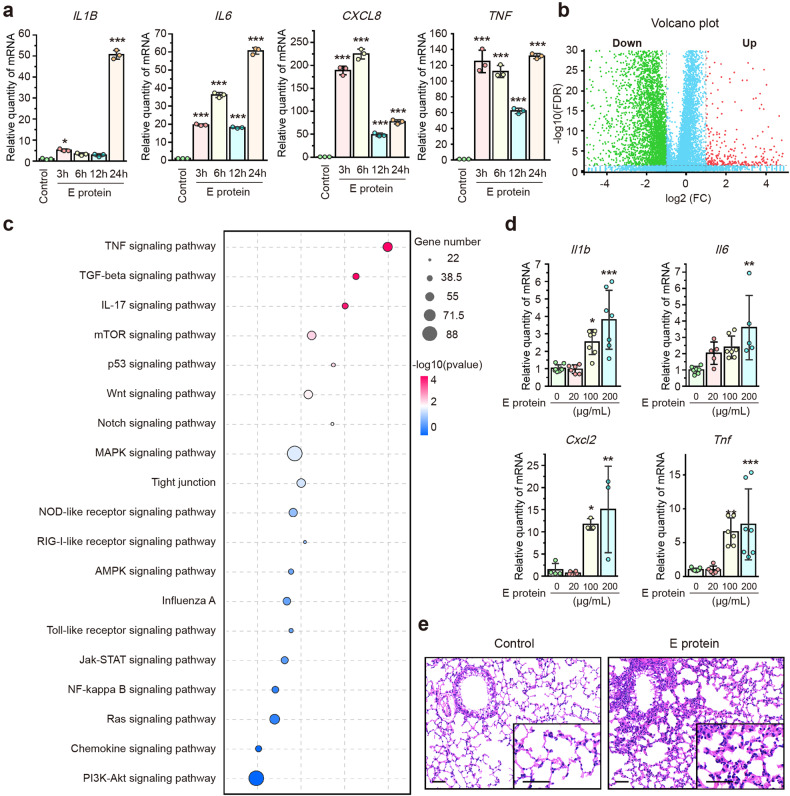
SARS-CoV-2 envelope (E) protein induced robust inflammation in airway epithelial cells. **a** Quantitative real-time PCR analyses showing the expression of pro-inflammatory cytokines in BEAS-2B cells stimulated with E protein (50 μg/ml) for the indicated time (*n* = 3). **b** A volcano plot from RNA sequencing analysis illustrating the distribution of all differentially expressed genes (DEGs) in BEAS-2B cells with and without E protein (50 μg/ml) stimulation. **c** Kyoto Encyclopedia of Genes and Genomes pathway enrichment analysis of DEGs in BEAS-2B cells stimulated with E protein (50 μg/ml) for 12 h. The dot size represents the number of DEGs, while the dot’s color corresponds to the *P* value. **d** Quantitative real-time PCR analyses showing the expression of pro-inflammatory cytokines or chemokines in the lung tissues of mice after intratracheal instillation of different concentrations of E protein for 24 h (*n* = 3–9). Data are shown as means ± S.D. ns = no significant, ^*^P < 0.05, ^**^P < 0.01, ^***^P < 0.001 versus the control group. **e** Hematoxylin and eosin (HE) staining of lung samples from mice after intratracheal instillation of E protein (100 μg/ml) for 24 h. Scale bar = 50 μm

The E protein of SARS-CoV has been reportedly associated with heightened inflammatory responses.^31,32^ We next tested whether SARS-CoV-2 E protein could elicit airway epithelial inflammation. After incubation with E protein, but not the solvents per se, with airway epithelial cells for various durations (3, 6, 12 and 24 h), the expression levels of pro-inflammatory cytokines/chemokines [including interleukin-1β (IL-1β), IL-6, IL-8 and tumor necrosis factor-α (TNF-α)] were markedly up-regulated (Fig. 2a and Supplementary Fig. 5c, h), which mirrored the levels reported in the serum of COVID-19 patients.^33^ To gain a comprehensive understanding of the molecular effects of E protein on airway epithelial cells, we conducted RNA-seq analysis, revealing significant regulation of numerous DEGs by E protein (Fig. 2b). These DEGs were significantly enriched in the immune-related pathways, such as the TNF signaling pathway, mitogen-activated protein kinase (MAPK) signaling pathway and NF-κB signaling pathway, as determined by Kyoto Encyclopedia of Genes and Genomes (KEGG) pathway analysis (Fig. 2c). To validate these results in an in vivo setting, we established a murine model of E protein stimulation via intratracheal instillation. E protein induced heightened pulmonary inflammation in a concentration-dependent manner (Fig. 2d), and elicited notable pulmonary leukocyte infiltration (Fig. 2e). Overall, these findings demonstrated that SARS-CoV-2 E protein triggered airway epithelial inflammation, both in vitro and in vivo.

### SARS-CoV-2 E protein induces airway inflammation via the TLR2/4-JNK-AP-1 pathway

**Fig. 3:**
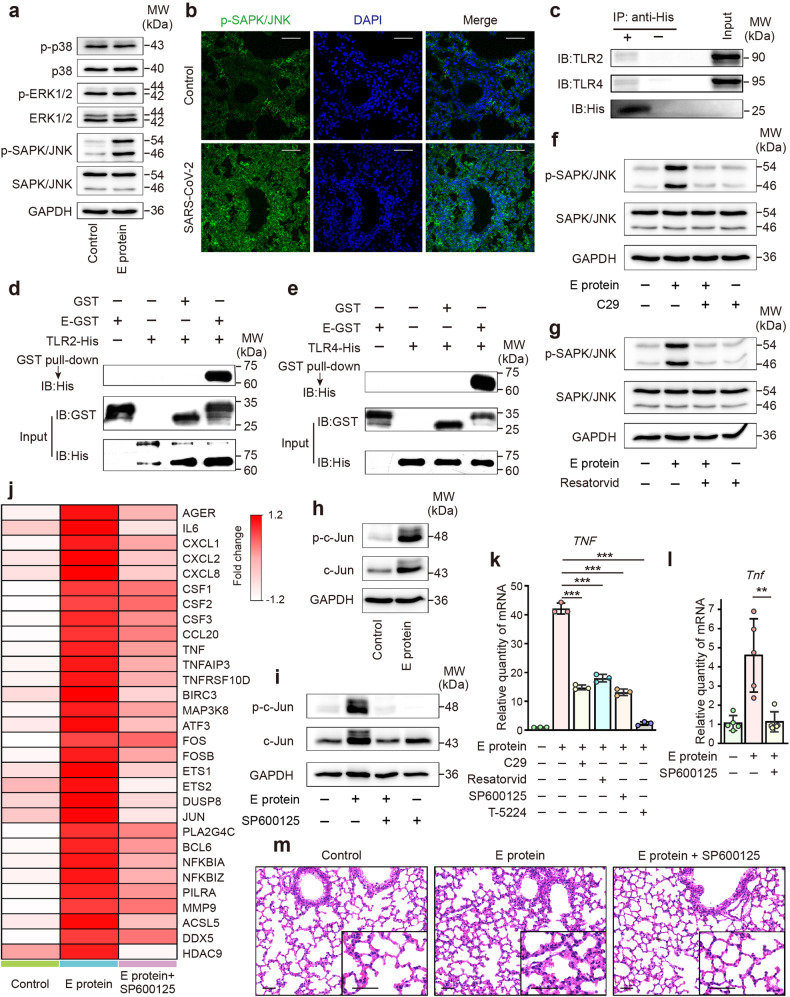
SARS-CoV-2 envelope (E) protein induced airway inflammation via the TLR2/4-JNK-AP-1 signaling pathway. **a** Representative immunoblots showing the phosphorylation level of p38, ERK and JNK in BEAS-2B cells after E protein (50 μg/ml) stimulation for 12 h. GAPDH served as a loading control. **b** Immunofluorescence staining of lung sections showing the phosphorylation of JNK in Ad5-hACE2 transgenic mice with or without SARS-CoV-2 infection (1 × 10^5^ PFU). Scale bar = 50 μm. **c** Co-immunoprecipitates and the total lysates (Input) were detected by Western Blotting using anti-TLR2 and anti-TLR4 antibody. **d** GST pull-down assays using GST-E protein and TLR2. TLR2 binding to GST-E protein was determined by using an anti-His antibody. **e** GST pull-down assays using GST-E protein and TLR4. TLR4 binding to GST-E protein was determined by using an anti-His antibody. **f** Representative immunoblots showing the effect of C29 (50 μM), an inhibitor of TLR2, on the phosphorylation level of JNK in BEAS-2B cells after stimulation with E protein (50 μg/ml) for 12 h. GAPDH served as a loading control. **g** Representative immunoblots showing the effect of Resatorvid (5 μM), an inhibitor of TLR4, on the phosphorylation level of JNK in BEAS-2B cells after stimulation with E protein (50 μg/ml) for 12 h. GAPDH served as a loading control. **h** Representative immunoblots showing the phosphorylation level of c-Jun in BEAS-2B cells after E protein (50 μg/ml) stimulation for 12 h. GAPDH served as a loading control. **i** Representative immunoblots showing the effect of SP600125 (10 μM), the selective inhibitor of JNK, on the phosphorylation level of JNK in BEAS-2B cells after stimulation with E protein (50 μg/ml) for 12 h. GAPDH served as a loading control. **j** Heatmap showing significant changes in mRNA expression of inflammation-related genes in BEAS-2B cells stimulated with E protein (50 μg/ml) with or without SP600125 (10 μM) for 12 h (*n* = 3/group). **k** Quantitative real-time PCR analyses showing the effect of C29 (50 μM), SP600125 (10 μM) and the selective AP-1 inhibitor T-5224 (10 μM) on the expression of tumor necrosis factor-α (TNF-α) in BEAS-2B cells stimulated with E protein (50 μg/ml) for 12 h (*n* = 3). **l** Quantitative real-time PCR analyses showing the effect of SP600125 (30 mg/kg) on the expression of TNF-α in the lung samples from mice after intratracheal instillation of E protein (100 μg/ml) for 24 h (*n* = 5). Data are shown as means ± S.D. ^**^P < 0.01, ^***^P < 0.001 indicated by lines. **m** Hematoxylin and eosin (HE) staining of lung samples from mice after intratracheal instillation of E protein (100 μg/ml) for 24 h, with or without intraperitoneal pre-treatment of SP600125 (30 mg/kg). Scale bar = 50 μm

We next examined the activation of MAPK signaling triggered by SARS-CoV-2 E protein. The JNK signaling pathway, but not p38 or ERK, was activated after E protein stimulation in airway epithelial cells (Fig. 3a). Furthermore, the augmented phosphorylation of JNK was also observed in the murine model of SARS-CoV-2 infection (Fig. 3b). JNK can be activated by a range of pattern recognition receptors, of which the TLRs have been extensively studied.^27^ Because TLR2 and TLR4 are related to the recognition of viral proteins,^34^ and TLR2 reportedly acted as the receptor for SARS-CoV-2 E protein,^26^ we next interrogated whether TLR2 and TLR4 were implicated in the activation of JNK signaling triggered by E protein. Co-IP technique (Fig. 3c) and GST pull-down assay (Fig. 3d, e) confirmed the interactions of E protein with both TLR2 and TLR4. Furthermore, the E protein-stimulated JNK phosphorylation was significantly attenuated by the TLR2 inhibitor C29 (Fig. 3f) and the TLR4 inhibitor Resatorvid (Fig. 3g).

We next sought to identify the downstream signaling molecules of JNK in E protein-stimulated airway epithelial cells. Interestingly, E protein augmented the phosphorylation of c-Jun (Fig. 3h), a key component of transcription factor activator protein-1 (AP-1), which could be abolished by the selective JNK inhibitor SP600125 (Fig. 3i). We then investigated whether JNK was implicated in airway epithelial inflammation induced by E protein. Heatmap of DEGs in Fig. 3j showed that SP600125 suppressed the inflammation-related genes which were up-regulated by E protein in airway epithelial cells. Additionally, inhibiting TLR2, TLR4, JNK or AP-1 resulted in the down-regulation of pro-inflammatory cytokines/chemokines (Fig. 3k and Supplementary Fig. 6a). The anti-inflammatory effect of SP600125 was further validated in mice intratracheally instilled with E protein (Fig. 3l, m and Supplementary Fig. 6b). Taken together, the TLR2/4-JNK-AP-1 signaling pathway mediated the exuberant inflammatory response triggered by E protein in airway epithelial cells.

### SARS-CoV-2 E protein induces elevated [Cl^−^]_i_ through TLR2/4-JNK-AP-1 pathway in airway epithelial cells

**Fig. 4:**
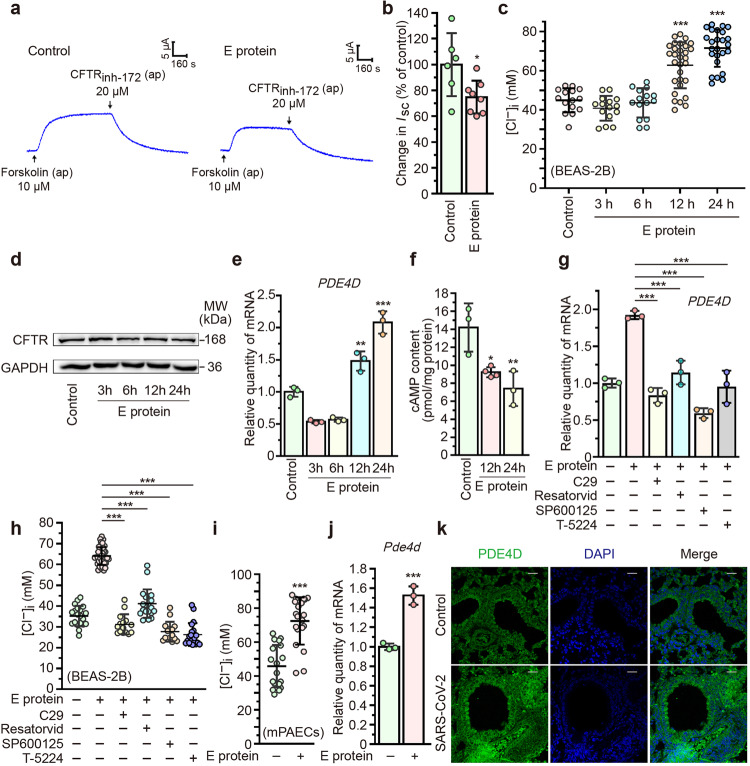
SARS-CoV-2 envelope (E) protein induced elevated intracellular Cl^−^ concentration ([Cl^−^]_i_) through TLR2-JNK-AP-1 pathway in airway epithelial cells. **a** Representative trace showing the short-circuit current (*I*_SC_) response induced by apical (ap) administration of forskolin (10 μM) with (right panel) or without (left panel) E protein (50 μg/ml) stimulation for 24 h in 16HBE14o- cells. **b** The effect of E protein (50 μg/ml) on the *I*_SC_ currents elicited by forskolin (10 μM, ap) in 16HBE14o- cells. (*n* = 6–8). **c** [Cl^−^]_i_ was measured in BEAS-2B cells stimulated with E protein (50 μg/ml) for the indicated time points. (*n* = 15–30 cells for each group). **d** Representative immunoblots showing the expression level of cystic fibrosis transmembrane conductance regulator (CFTR) in BEAS-2B cells after E protein (50 μg/ml) stimulation for the indicated time points. GAPDH served as a loading control. **e** Quantitative real-time PCR analyses showing the expression of phosphodiesterase 4D (PDE4D) in BEAS-2B cells stimulated with E protein (50 μg/ml) for the indicated time points (*n* = 3). **f** Effect of E protein (50 μg/ml) on the intracellular level of cAMP in BEAS-2B cells (*n* = 3). **g** Effects of the TLR2 inhibitor C29 (50 μM), the TLR4 inhibitor Resatorvid (5 μM), the JNK inhibitor SP600125 (10 μM) and the AP-1 inhibitor T-5224 (10 μM) on the expression of PDE4D in BEAS-2B cells after E protein (50 μg/ml) stimulation for 12 h (*n* = 3). **h** [Cl^−^]_i_ was measured in BEAS-2B cells stimulated with E protein (50 μg/ml) for 12 h, with or without pre-treatment with C29 (50 μM), Resatorvid (5 μM), SP600125 (10 μM) or T-5224 (10 μM). (*n* = 15–40 cells for each group). **i** [Cl^−^]_i_ was measured in primary cultured mouse airway epithelial cells (mPAECs) stimulated with E protein (50 μg/ml) for 24 h (*n* = 18–20 cells for each group). **j** Quantitative real-time PCR analyses showing the expression of PDE4D in the mPAECs stimulated with E protein (100 μg/ml) for 24 h (*n* = 3). Data are shown as means ± S.D. ns = no significant, ^*^P < 0.05, ^**^P < 0.01, ^***^P < 0.001 versus the control group or indicated by lines. **k** Immunofluorescence images showing the expression of PDE4D in Ad5-hACE2 transgenic mice with or without SARS-CoV-2 infection (1 × 10^5^ PFU). Scale bar = 50 μm

In light of the growing evidence supporting the pathogenic role of intracellular Cl^−^ in promoting inflammation,^22,35,36^ we investigated the effect of SARS-CoV-2 E protein on Cl^−^ transport across airway epithelium. Ussing chamber experiments revealed that E protein significantly attenuated the forskolin-elicited short circuit current (*I*_SC_) response in airway epithelial cells (Fig. 4a, b). This finding indicated impaired transepithelial Cl^−^ secretion, which is typically mediated by CFTR, a pivotal Cl^−^ channel on airway epithelial cells. Consistently, E protein markedly increased [Cl^−^]_i_ at 12 and 24 h in airway epithelial cells (Fig. 4c, i), indicating perturbed intracellular Cl^−^ homeostasis during SARS-CoV-2 infection. To explore the mechanism underlying the elevated [Cl^−^]_i_, we investigated the effect of E protein on CFTR expression and function. Intriguingly, E protein did not alter the expression of CFTR (Fig. 4d) or interaction with CFTR (Supplementary Fig. 7). Previously, we have demonstrated that the LPS-mediated elevation of [Cl^−^]_i_ in airway epithelium depended on the phosphodiesterase 4 (PDE4)-cAMP pathway, which modulated the activity of CFTR.^35^ We thus examined the effect of E protein on the expression of PDE4D in airway epithelial cells. As shown in Fig. 4e, PDE4D expression was significantly up-regulated after 12 and 24 h of stimulation with E protein. Additionally, decreased intracellular cAMP content was observed after stimulation with E protein (Fig. 4f). In vivo studies in primary cultured mouse airway epithelial cells (mPAECs) and a murine model of SARS-CoV-2 infection also revealed increased [Cl^−^]_i_ and PDE4D expression (Fig. 4i–k). Finally, we investigated the mechanism underlying the disturbance of PDE4-Cl^−^ signaling, demonstrating that pre-treatment with the inhibitors of TLR2, TLR4, JNK or AP-1 significantly reversed the up-regulated expression of PDE4D (Fig. 4g) and increased [Cl^−^]_i_ (Fig. 4h) in the E protein-treated airway epithelial cells. These findings collectively demonstrated that SARS-CoV-2 E protein impaired CFTR-mediated Cl^−^ transport and elicited aberrant [Cl^−^]_i_ in airway epithelial cells through a TLR2/4-JNK-AP-1 dependent mechanism.

### SARS-CoV-2 E protein induces Cl^−^-driven airway inflammation via activating SGK1

**Fig. 5:**
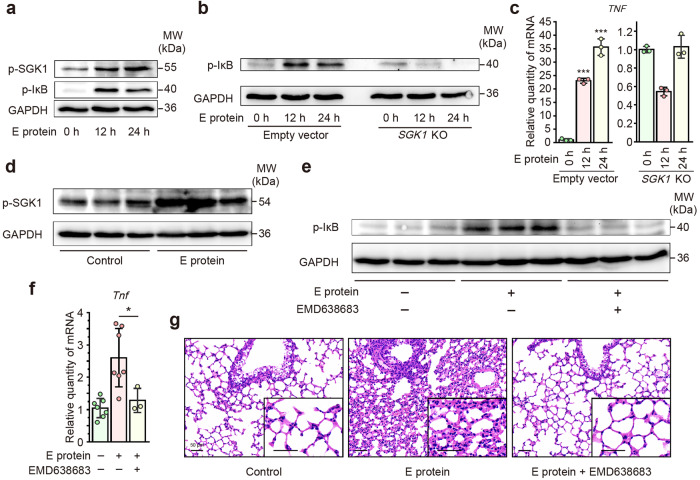
SARS-CoV-2 envelope (E) protein induced Cl^−^-driven airway inflammation via activating serum/glucocorticoid regulated kinase 1 (SGK1). **a** Representative immunoblots showing the phosphorylation level of IκB and SGK1 in BEAS-2B cells after E protein (50 μg/ml) stimulation for the indicated time points. GAPDH served as a loading control. **b** Representative immunoblots showing the effect of *SGK1* knockout (KO) on the phosphorylation level of IκB in BEAS-2B cells after E protein (50 μg/ml) stimulation for the indicated time points. GAPDH served as a loading control. **c** Quantitative real-time PCR analyses showing the effect of *SGK1* KO on the expression of tumor necrosis factor-α (TNF-α) in BEAS-2B cells stimulated with E protein (50 μg/ml) for the indicated time points (*n* = 3). **d** Representative immunoblots showing the phosphorylation level of SGK1 in lung samples derived from mice after intratracheal instillation of E protein (100 μg/ml) for 24 h. GAPDH served as a loading control. **e** Representative immunoblots showing the effect of EMD638683 (10 mg/kg), a selective inhibitor of SGK1, on the phosphorylation level of IκB in lung samples derived from mice after intratracheal instillation of E protein (100 μg/ml) for 24 h. GAPDH served as a loading control. **f** Quantitative real-time PCR analyses showing the effect of EMD638683 (10 mg/kg) on the expression of TNF-α in the lung samples from mice after intratracheal instillation of E protein (100 μg/ml) for 24 h (*n* = 3–8). Data are shown as means ± S.D. ^*^P < 0.05, ^***^P < 0.001 versus the control group or indicated by lines. **g** Hematoxylin and eosin (HE) staining of lung samples from mice after intratracheal instillation of E protein (100 μg/ml) for 24 h, with or without intraperitoneal pre-treatment of EMD638683 (10 mg/kg). Scale bar = 50 μm

SGK1 is a ubiquitously expressed serine/threonine kinase known to mediate NF-κB activation and inflammation.^37^ We have previously identified SGK1 as a novel Cl^−^-sensitive kinase.^35^ Therefore, we aimed to verify whether SGK1 is implicated in the airway inflammatory responses elicited by E protein. Western blot assays demonstrated that E protein stimulation led to the simultaneous phosphorylation of IκB and SGK1 (Fig. 5a). To further investigate the specific role of SGK1, we utilized SGK1 knockout cells as compared with the empty vector control. Knock-out of SGK abrogated the enhanced phosphorylation of IκB and the up-regulation of pro-inflammatory cytokines/chemokines induced by E protein (Fig. 5b, c and Supplementary Fig. 8a). These findings were also consistent in the in vivo murine model, where E protein stimulation resulted in increased SGK1 phosphorylation (Fig. 5d). Furthermore, pre-treatment with EMD638683, a selective inhibitor of SGK1 administered intraperitoneally, attenuated the pulmonary inflammation induced by E protein in mice (Fig. 5e–g and Supplementary Fig. 8b). Taken together, these results suggested the Cl^−^-SGK1 signaling pathway is implicated in the airway epithelial inflammation triggered by SARS-CoV-2 E protein.

### PDE4 inhibitor protects against E protein-induced airway inflammation through suppressing intracellular Cl^−^ accumulation

**Fig. 6:**
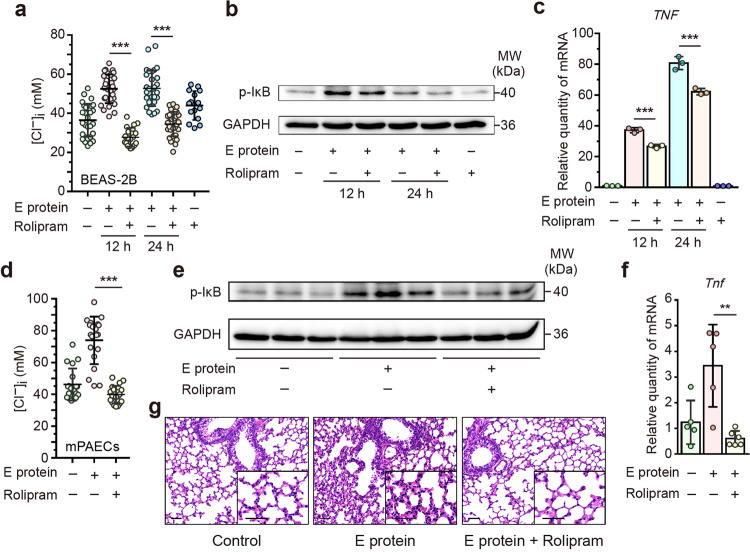
Phosphodiesterase 4 (PDE4) inhibitor protected against SARS-CoV-2 envelope (E) protein-induced airway inflammation through suppressing intracellular Cl^−^ accumulation. **a** Intracellular Cl^−^ concentration ([Cl^−^]_i_) was measured in BEAS-2B cells stimulated with E protein (50 μg/ml) for the indicated time points, with or without pre-treatment of rolipram (20 μM), a selective PDE4 inhibitor (*n* = 21–30 cells for each group). **b** Representative immunoblots showing the effect of rolipram (20 μM) on the phosphorylation level of IκB in BEAS-2B cells after E protein (50 μg/ml) stimulation for the indicated time points. GAPDH served as a loading control. **c** Quantitative real-time PCR analyses showing the effect of rolipram (20 μM) on the expression of tumor necrosis factor-α (TNF-α) in BEAS-2B cells stimulated with E protein (50 μg/ml) for the indicated time points (*n* = 3). **d** [Cl^−^]_i_ was measured in the primary cultured mouse airway epithelial cells (mPAECs) stimulated with E protein (50 μg/ml) for 24 h, with or without pre-treatment of rolipram (20 μM) (*n* = 19–20 cells for each group). **e** Representative immunoblots showing the effect of rolipram (10 mg/kg) on the phosphorylation level of IκB in lung samples from mice after intratracheal instillation of E protein (100 μg/ml) for 24 h. GAPDH served as a loading control. **f** Quantitative real-time PCR analyses showing the effect of rolipram (10 mg/kg) on the expression of TNF-α in the lung samples derived from mice after intratracheal instillation of E protein (100 μg/ml) for 24 h (*n* = 5–6). Data are shown as means ± S.D. ^**^P < 0.01, ^***^P < 0.001 indicated by lines. **g** Hematoxylin and eosin (HE) staining of the lung samples from mice after intratracheal instillation of E protein (100 μg/ml) for 24 h, with or without intraperitoneal pre-treatment of rolipram (10 mg/kg). Scale bar = 50 μm

In light that E protein increased [Cl^−^]_i_ in a PDE4-cAMP dependent fashion, we investigated the potential protective effects of agents targeting PDE4 against E protein-mediated elevation in [Cl^−^]_i_ and airway inflammation. Rolipram, a selective inhibitor of PDE4, abolished the increased [Cl^−^]_i_ elicited by E protein in both human airway epithelial cells (Fig. 6a) and mPAECs (Fig. 6d). Moreover, rolipram treatment attenuated the phosphorylation of IκB and the up-regulation of pro-inflammatory cytokines/chemokines in airway epithelial cells (Fig. 6b, c and Supplementary Fig. 9a). Importantly, in a murine model of SARS-CoV-2 E protein stimulation via intratracheal instillation, rolipram administration inhibited elevated [Cl^−^]_i_ in mPAECs and exerted anti-inflammatory effects in lung tissues (Fig. 6e–g and Supplementary Fig. 9b). These findings suggest that pharmacological interventions targeting at PDE4 inhibition may effectively alleviate intracellular Cl^−^ accumulation within the airway epithelial cells and mitigate the ongoing airway inflammation induced by SARS-CoV-2 infection.

### Validation of the effects of E protein on primary cultured human airway epithelial cells (hPAECs)

**Fig. 7:**
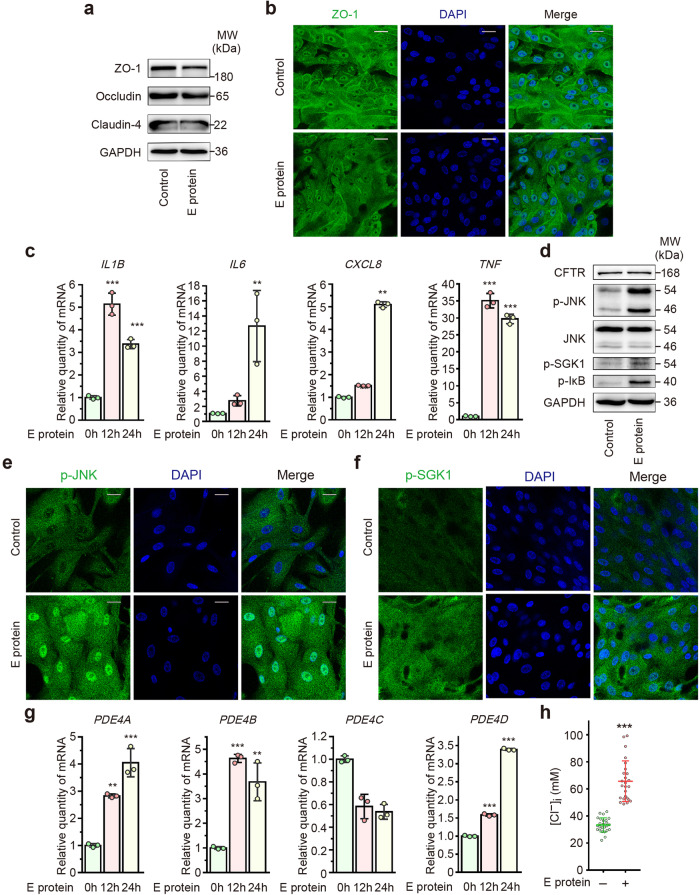
SARS-CoV-2 envelope (E) protein down-regulated tight junction proteins and triggered inflammation and intracellular Cl^−^ accumulation in human primary cultured airway epithelial cells (hPAECs). **a** Representative immunoblots showing the expression level of ZO-1, occludin and claudin-4 in hPAECs stimulated with E protein (50 μg/ml) for 6 h. GAPDH served as a loading control. **b** Immunofluorescence images showing the expression of ZO-1 in hPAECs, in the absence or presence of E protein stimulation for 6 h. **c** Quantitative real-time PCR analyses showing the expression of pro-inflammatory cytokines in hPAECs stimulated with E protein (50 μg/ml) for 12 h (*n* = 3). **d** Representative immunoblots showing the expression of cystic fibrosis transmembrane conductance regulator (CFTR) and the phosphorylation level of JNK, serum/glucocorticoid regulated kinase 1 (SGK1) and IκB in hPAECs after stimulation with the E protein (50 μg/ml) for 12 h. GAPDH served as a loading control. **e** Immunofluorescence images showing the phosphorylation of JNK in hPAECs, in the absence or presence of E protein (50 μg/ml) stimulation. **f** Immunofluorescence images showing the phosphorylation of SGK1 in hPAECs, in the absence or presence of E protein (50 μg/ml) stimulation. **g** Quantitative real-time PCR analyses showing the expression of phosphodiesterase 4 (PDE4) in hPAECs stimulated with E protein (50 μg/ml) for the indicated different time points (*n* = 3). **h** Intracellular Cl^−^ concentration ([Cl^−^]_i_) was measured in hPAECs stimulated with E protein (50 μg/ml) for 12 h (*n* = 25 cells for each group). Data are shown as means ± S.D. ^**^P < 0.01, ^***^P < 0.001 versus the control group

To validate the aforementioned findings, we finally utilized the hPAECs model. Interestingly, E protein down-regulated the expression of ZO-1 and occludin in hPAECs, while the expression of claudin-4 remained unaffected (Fig. 7a, b and Supplementary Fig. 10). Additionally, E protein triggered an excessive inflammatory response, characterized by increased expression of pro-inflammatory cytokines (Fig. 7c) and heightened phosphorylation of JNK, IκB and SGK1 (Fig. 7d–f) in hPAECs. Consistent with our observations in human airway epithelial cell lines, E protein did not alter the expression of CFTR, but instead augmented the expression of PDE4 subtypes (Fig. 7g), leading to aberrant intracellular Cl^−^ accumulation in hPAECs (Fig. 7h). These results further confirmed the role of SARS-CoV-2 E protein in disrupting airway epithelial integrity, promoting inflammatory responses, and disturbing intracellular Cl^−^ homeostasis in airway epithelial cells.

## Discussion

E protein is a crucial structural component of SARS-CoV-2 implicated in viral infection, although the precise mechanism remains to be elucidated. Here, we found that SARS-CoV-2 E protein significantly down-regulated the expression of tight junction-associated proteins and elicited airway epithelial inflammation. Furthermore, we unraveled the proinflammatory properties of SARS-CoV-2 E protein through elevating [Cl^−^]_i_ and activating downstream Cl^−^ signaling, which was dependent on the activation of TLR2/4-JNK-AP-1 pathway. These findings may provide important clues as to how SARS-CoV-2 may trigger airway epithelial injury and provoke excessive inflammatory responses through the involvement of E protein.

The airway epithelial tight junction serves as a crucial physical barrier, safeguarding against microbial infection, through a constellation of tight junctional proteins such as ZO-1, occludin, and claudins.^29^ SARS-CoV-2 reportedly elicited airway epithelial damage, evidenced by the disrupted expression of ZO-1.^15^ Similar observations have been made in the context of other respiratory virus infections, such as rhinovirus, where disruption of tight junctions and loss of tight junctional proteins have been documented.^38^ Here, we presented the evidence supporting the role of SARS-CoV-2 E protein in down-regulating tight junction protein expression and impairing the integrity of airway epithelium, which in turn facilitated the invasion of bacteria such as *P. aeruginosa*. Our study confirmed the pivotal role of E protein in the pathophysiology of airway epithelial damage during SARS-CoV-2 infection, particularly in the context of co-infection with other bacterial pathogens. Interestingly, our results revealed the different expression patterns between the primary cultured airway epithelial cells and the immortalized airway epithelial cell lines after E protein stimulation. Apart from epithelial-barrier functions, the tight junction proteins have also played a central role in controlling epithelial proliferation and differentiation in vertebrates,^39^ which may contribute to the discrepancy observed between primary cells and immortalized cells. Additionally, the tight junction pores varied among different cell types.^40^ Given that the primary cells are composed of multiple cell subtypes, the discrepancy observed in our study may be attributable to the difference in cell types.

As a core structural component, E protein from various coronaviruses has been shown to elicit inflammatory responses in human.^32^ For SARS-CoV, E protein has been found to activate NLRP3 inflammasome, leading to the secretion of mature IL-1β.^41^ Similarly, a previous study has indicated that SARS-CoV-2 E protein also triggers NLRP3 inflammasome activation in the later stages of infection.^42^ Moreover, another study suggested that SARS-CoV-2 E protein might stimulate NF-κB activation by interacting with TLR2 and subsequently activating the downstream signaling pathways in macrophages.^26^ Thus, the underlying mechanism of airway epithelial inflammation elicited by coronavirus E protein warrants further investigation. While SARS-CoV E protein has been shown to up-regulate pro-inflammatory cytokine expression in a p38 MAPK dependent manner,^31^ our study demonstrated that SARS-CoV-2 E protein elicited airway inflammatory responses through the activation of JNK signaling, which occurs downstream of binding with both TLR2 and TLR4. These findings highlight the possibility that E protein of both SARS-CoV-2 and SARS-CoV may trigger inflammatory responses through distinct signaling pathways. As another important surface molecules of SARS-CoV-2, S protein was reported to activate TLR2^9^ and TLR4^43^ in macrophages. Intriguingly, the impaired airway epithelial barrier or the aberrant PDE4-CFTR-Cl^−^ signaling were not observed in airway epithelial cells stimulated with S protein (Supplementary Fig. 11), although the phosphorylation of JNK was slightly augmented. These findings provide valuable insights into the differing pathogenic roles of the E protein and S protein.

In mammalian cells, [Cl^−^]_i_ is dynamically regulated by multiple Cl^−^ channels and transporters.^20^ Of these, CFTR is a cAMP-activated Cl^−^ channel located on the apical membrane of airway epithelium, playing a pivotal role in mediating transepithelial ion transport and mucociliary clearance.^44^ The dysfunction of CFTR diminished Cl^−^ and HCO_3_^−^ secretion, resulting in mucus retention and promoting the colonization of pathogens, leading to chronic airway inflammation.^44,45^ Moreover, CFTR has been shown to down-regulate the activity of NF-κB,^46^ suggesting that it is a core modulator of airway inflammation.^47^ Interestingly, our study revealed that E protein did not directly interact with CFTR or alter its expression, therefore other mechanisms might be implicated in the proinflammatory events. We have further demonstrated that E protein stimulation elicited the up-regulation of PDE4D, a major PDE4 family member responsible for cAMP hydrolysis,^48^ which was dependent on TLR2/4-JNK-AP-1 signaling. As a consequence, intracellular cAMP levels were markedly reduced. Considering that cAMP-dependent protein kinase (PKA)-mediated phosphorylation is essential for CFTR activation,^49,50^ the decreased cAMP concentration induced by E protein could hinder the opening of CFTR, contributing to the elevated [Cl^−^]_i_ within the airway epithelial cells. Similar observations have been made in previous studies with structural components of influenza virus, such as hemagglutinin^51^ and matrix-2 protein,^52^ where impaired CFTR activity was associated with viral infection. Pathogenic mutations in CFTR cause cystic fibrosis (CF), a monogenic disease manifesting as chronic airway inflammation. Notably, compared with 16HBE14o- cell line which was heterologous for CFTR, the E protein could slightly augment the phosphorylation of JNK in a TLR2-dependent manner in CFBE41o- cells, a human CF bronchial epithelial cell line which expressed the almost undetectable fully glycosylated mature form of CFTR^53^ (Supplementary Fig. 12). In light that SARS-CoV-2 infection in CF patients was relatively minor, and the virus did not seem to have a significant impact on the severity of CF,^54^ our results indicated the involvement of CFTR-Cl^−^ signaling in the pathogenesis of both CF and COVID-19. Interestingly, our findings also indicated that E protein from both SARS-CoV (Supplementary Fig. 13) and SARS-CoV-2 could trigger intracellular Cl^−^ accumulation, suggesting a potential common mechanism for excessive airway inflammation during infection caused by these coronaviruses. In this context, our study highlights the potential of PDE4 inhibitors (such as roflumilast which has been shown to provide clinical benefits in patients with chronic obstructive pulmonary disease), as a candidate treatment option for alleviating intracellular Cl^−^ accumulation and mitigating inflammatory responses induced by SARS-CoV-2 E protein in COVID-19 cases.

Cl^−^ is the principal anion within the cells, playing crucial roles in modulating transepithelial water and electrolyte transportation, cell volume changes and cell excitability.^20,55^ Beyond its fundamental physiological functions, Cl^−^ can also act as a signaling effector by regulating the expression of Cl^−^-dependent genes or activity of target proteins,^21^ such as with no lysine kinase 1 (WNK1)^56^ and WNK4.^57^ Notably, our previous research has revealed that SGK1 functioned as a novel Cl^−^-sensing kinase, with higher Cl^−^ levels leading to an up-regulation of SGK1 activity.^35^ In our study, we have further demonstrated that SARS-CoV-2 E protein stimulation resulted in a substantial phosphorylation of SGK1, both in vitro and in vivo. Furthermore, both genetic deletion and pharmacological inhibition of SGK1 yielded significant attenuation of IκB phosphorylation, leading to suppression of pro-inflammatory cytokines and chemokine induced by E protein. These findings suggest that SGK1 may represent a promising novel therapeutic target for SARS-CoV-2 infection. Previous studies have indicated that SGK1 promotes the phosphorylation and acetylation of NF-κB, thereby activating NF-κB through the phosphorylation of both IKKα and p300.^37^ Consistent with our previous findings in airway inflammation induced by SARS-CoV-2 N protein,^11^ *P. aeruginosa* LPS^35^ and *Toxoplasma gondii*,^58^ the present study has further underscored the role of augmented SGK1 phosphorylation as a consequential response to pathogen infection in modulating immune and inflammatory responses.

Recombinant protein has important applications in biomedical research, clinical diagnostics and therapeutics. Besides, recombinant protein is valuable to explore the cell signaling pathways. However, several issues may arise in the experimental settings of recombinant protein, such as incorrect folding and incorrect modification of the protein after translation.^59^ More attention needs to be paid to improving the experimental techniques. It should be noted that the recombinant E protein used in our study contained a β-barrel protein domain. To exclude the potential non-specific effect, we attempted to verify our findings by generating SARS-CoV-2 E-expressed airway epithelial cells using the Gene ORF cDNA clone expression plasmid of SARS-CoV-2 E protein. The results revealed the positive expression of E protein, as well as the co-localization of E protein and the ER marker calreticulin in airway epithelial cells (Supplementary Fig. 14a and b). Interestingly, endogenous expression of E protein could trigger up-regulation of the pro-inflammatory cytokines (Supplementary Fig. 14c), which further demonstrating the inducible role of E protein in airway epithelium.

**Fig. 8:**
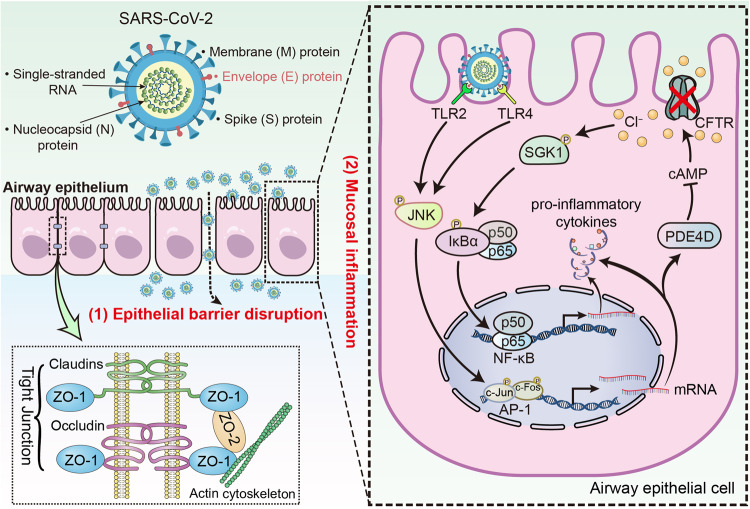
Schematic diagram showing the pathophysiological role of SARS-CoV-2 envelope (E) protein in airway epithelial cells. SARS-CoV-2 E protein down-regulated tight junctional proteins, impairing airway epithelial integrity. E protein also activated TLR2/4-JNK-AP-1 signaling, leading to increased PDE4 expression and reduced cAMP levels. This disruption of cAMP signaling impaired CFTR-mediated Cl^−^ transport, resulting in elevated [Cl^−^]_i_ and activation of SGK1, which contributes to the ongoing inflammation in the airway epithelium

In summary, SARS-CoV-2 E protein down-regulated tight junctional proteins, compromising the integrity of the airway epithelial barrier. Moreover, E protein markedly decreased intracellular cAMP levels through up-regulating PDE4 expression via TLR2/4-JNK-AP-1 signaling, leading to the impaired CFTR-mediated Cl^−^ transport. Consequently, the elevation of [Cl^−^]_i_ may modulate the ongoing inflammation by promoting the phosphorylation of SGK1 within the airway epithelium (Fig. 8). Our findings provide a novel perspective for the role of E protein in promoting the accumulation of Cl^−^ levels within the airway epithelial cells, thus exacerbating the inflammatory responses. Therapeutic interventions aimed at increasing cAMP levels to open the CFTR, or using CFTR agonizts and SGK1 suppressors to abrogate the intracellular inflammatory cascade, may hold promise for mitigating the airway inflammation induced by SARS-CoV-2.

## Materials and methods

### Peptides and reagents

Recombinant SARS-CoV-2 E Protein was purchased from Novoprotein (Shanghai, China), and the target gene was expressed with a β-barrel protein platform and 6 × His tag at the N-terminus,^60^ and dissolved in the solution of 20 mM Tris-HCl and 200 mM NaCl. SARS-CoV-2 Spike S1 and S2 recombinant protein were purchased from Sino Biological (Beijing, China). SARS-CoV E protein was purchased from Glpbio (USA); rolipram from Sigma Aldrich (USA); C29, Resatorvid, SP600125, T-5224 and EMD638683 from MedChemExpress (MCE, USA).

### Cell culture

The human bronchial epithelial cell line BEAS-2B and 16HBE14o- were cultured as previously described.^35,58^ Briefly, BEAS-2B cells, 16HBE14o- cells, and CFBE41o- cells were cultured in high glucose Dulbecco’s modified Eagle medium (DMEM, Hyclone, USA) or Minimum Essential Medium (MEM, Corning, USA) supplemented with 1% (vol/vol) penicillin−streptomycin (Hyclone, USA) and 10% (vol/vol) fetal bovine serum (Gibco, USA), maintaining in 5% CO_2_ at 37 °C. Cells were cultured to 80% confluence, and serum-starved overnight before E protein stimulation. BEAS-2B cells were inoculated in the 24-well cell culture plate and stimulated with 400 μL SARS-CoV-2 E protein (50 μg/mL) for 12 h. After stimulation, the E protein could be observed in cell lysates and was co-localized with ER marker calreticulin (Supplementary Fig. 5d, e). For additional experiments, cells were pre-treated with several inhibitors for 1 hr before E protein challenge. Besides, SGK1 gene knockout (KO) BEAS-2B cells and empty vector control BEAS-2B cells were constructed as previously described.^35^

### TER measurement

TER values of epithelial cell monolayers were measured to evaluate the tight junction permeability.^61^ Briefly, 16HBE14o- cells were seeded into plate inserts (12 mm diameter, 3.0 μm pore size, BIOFIL JET), and the TER of 16HBE14o- monolayers was measured in the presence or absence of SARS-CoV-2 E protein or S protein, using the Millicell-ERS Electrical Resistance System (Millipore, USA). The TER values were obtained by subtracting the intrinsic resistance of the membrane, and multiplied by the surface area of the filter (1.12 cm^2^). After stimulation, the E protein significantly decreased the TER at a concentration of 50 μg/mL (Supplementary Fig. 5f).

### Cell permeability assay

FD4 was used to evaluate the paracellular permeability of airway epithelium.^62^ Briefly, 16HBE14o- cells were cultured in plate inserts (3.0 μm pore size) until a confluent monolayer was formed. FD4 (Sigma, USA) at a concentration of 1 mg/ml was added to the upper chambers accompanied with or without SARS-CoV-2 E protein (50 μg/mL) stimulation. The fluorescence intensity of FD4 passage at indicated times was detected by fluorescence microplate reader (Synergy H1, BioTek, USA), with excitation and emission wavelengths at 490 nm and 520 nm, respectively. The fluorescence intensity of serial standard dilutions ranging from 0–50 μg/mL was also obtained from the fluorescence reader and a standard curve was made to determine the FD4 concentration of the samples. After stimulation, the E protein resulted in a significant increase in the paracellular flux of FD4 at a concentration of 50 μg/mL (Supplementary Fig. 5g).

### Bacterial culture and monolayer invasion assay

*Pseudomonas aeruginosa* strain PAO1 was by courtesy of Dr. Lei Ni (Shenzhen Institute of Advanced Technology, Chinese Academy of Sciences, Shenzhen, P. R. China). Bacteria were grown on solid Luria–Bertani (LB) medium (1.5% agar) at 37 °C for 18 h and bacterial colonies were scraped off and routinely grown in LB broth at 37 °C with shaking (200 rpm) overnight. Bacterial cultures were resuspended in MEM medium with no antibiotics until an optical density at a wavelength of 600 nm was 1.0, corresponding to ~1×10^9^ colony-forming units (CFU) per ml.

The penetration of PAO1 through the 16HBE14o- cell monolayer was evaluated to determine the effect of SARS-CoV-2 E protein on the epithelial tight junction permeability and the invasiveness of respiratory pathogens. Briefly, 16HBE14o- cells were seeded on plate inserts to form a monolayer with tight junctions, monitored by measurement of TER values. 16HBE14o- cells were then infected apically with PAO1 at a multiplicity of infection (MOI) of 20,^63^ with or without SARS-CoV-2 E protein stimulation. 2 h later, the PAO1 penetrated to the basolateral medium were counted through plating serially diluted medium onto LB agar plates.

### Measurement of bronchoalveolar epithelial permeability

Male ICR mice, weighing 25–30 g, were obtained from the Laboratory Animal Center of Sun Yat-sen University (Guangzhou, China). Mice were anesthetized with tribromoethanol [0.2 ml/10 g body weight of a 1.25% (vol/vol) solution] and intratracheally instilled with 50 μl E protein (200 μg/ml) or an equal volume of normal saline for 6 h. Mice were next injected intravenously via the tail vein with FD4 (5 mg/ml; 10 mg/kg body weight). 15 min later, the blood was collected and stored at 4 °C overnight, and the serum was isolated by centrifugation at 600 *g* for 5 min. Meanwhile, using a tracheal cannula, the BALF was collected via bronchoalveolar lavage for three times with 600 μl of phosphate buffered saline (PBS) per wash. The fluorescence intensity of serum (10-fold diluted by PBS) or BALF samples was detected as described above. Finally, the ratio of fluorescence in BALF and serum was calculated to assess pulmonary epithelial permeability. All procedures were performed in strict accordance with the animal use protocols approved by the Sun Yat-sen University Institutional Animal Care and Use Committee (Guangzhou, China).

### RNA sequencing

Total RNA was extracted by using TRIzol reagent (Invitrogen, USA) and the quality of RNA was assessed using agarose gel electrophoresis and Agilent 2100 Bioanalyzer (Agilent Technologies, USA). Next, cDNA libraries were constructed and sequenced on the Illumina HiSeq2500 platform by Gene Denovo Biotechnology (Guangzhou, China). Differentially expressed genes (DEGs) between two different groups were identified using DESeq2 software. A false discovery rate (FDR) below 0.05 and a log_2_ (fold change) ≥ 1 was considered significant.

### Quantitative real-time PCR

The total RNA extracted from airway epithelial cells or mouse lung samples by using *SteadyPure* Universal RNA Extraction Kit (Accurate Biology, China) was reverse-transcribed with HiScript II Q RT SuperMix for qPCR (+gDNA wiper) (Vazyme, China). Real-time PCR was performed using ChamQ SYBR qPCR Master Mix (Vazyme, China). The levels of target genes were normalized to that of GAPDH for cells, and HPRT1 for lung tissues, and the relative expression was calculated using the 2^-∆∆Ct^ algorithm, as described previously.^64^ The sequences of primers used in Real-Time PCR reactions are shown in Supplementary Table 1.

### Western blotting

The total protein was extracted from airway epithelial cells or mouse lung specimens by using radio immunoprecipitation assay (RIPA) lysis buffer (Beyotime, China). Western blotting analysis for the target proteins was performed as described previously.^64^ The primary antibody against ZO-1 (1:500, #61-7300) and Occludin (1:500, #71-1500) were purchased from Thermo Fisher Scientific (USA). The antibodies against p38 MAPK (1:1000, #8690), phospho-p38 MAPK (1:1000, #4511), p44/42 MAPK (Erk1/2, 1:1000, #4695), phospho-p44/42 MAPK (Erk1/2, 1:1000, #4370), SAPK/JNK (1:1000, #9252), phospho-SAPK/JNK (1:1000, #4668), c-Jun (1:1000, #9165), phospho-c-Jun (1:1000, #3270), and phospho-IκB-α (1:1000, #2859) were purchased from Cell Signaling Technology (USA). The antibody against phospho-SGK1 (1:1000, #36-002) was purchased from Sigma-Aldrich (USA). The antibodies against Claudin-4 (1:1000, #ab53156), TLR2 (1:1000, ab68159), CFTR (1:1000, #ab2784) and GAPDH (1:1000, #ab8245) were from Abcam (UK). The antibody against TLR4 (1:1000, #AF7017) was purchased from Affinity Biosciences (USA). The antibody against His-Tag (1:10000, #66005-1-Ig) was purchased from Proteintech (USA). The antibody against SARS-CoV-2 Envelope (1:1000, #GTX636915) was purchased from GeneTex (USA). The antibodies against SARS-CoV-2 Spike S1 (1:1000, #40591-R235) and Spike S2 (1:1000, #40590-T62) were from Sino Biological (China).

### Co-immunoprecipitation assay

Airway epithelial cells were lysed in cell lysis buffer for Western blotting and co-immunoprecipitation (Co-IP) assay (Beyotime, China) for 20 min. Cell lysates were next centrifuged at 12000 *g* for 5 min. The supernatant was incubated with 10 μg His-tagged E protein (Novoprotein, China) and Anti-His Tag Magnetic Beads (Abbkine, China) at 4 °C overnight on a rocking platform. The magnetic beads were, by using a magnetic separator, washed with the ice-cold PBS followed by removal of the supernatant. The collected magnetic beads were boiled for 5 min in the 1 × sodium dodecyl sulfate (SDS) loading buffer solution and Western blotting analysis was performed.

### Immunofluorescence staining

Airway epithelial cells were fixed with 4% paraformaldehyde for 20 min, and permeabilized with 0.1% Triton X-100 for 5 min. The mouse lungs were fixed in 10% formalin, embedded in paraffin and cut into 4 μm sections. Fixed cells or lung tissues were then blocked in 3% bovine serum albumin (BSA) for 1 hr, and incubated with anti-Envelope (GeneTex, #GTX636915 1:500), anti-calreticulin (Abcam, #ab22683, 1:200), anti-ZO-1 (Thermo Fisher Scientific, #61-7300, 1:50), anti-Occludin (Thermo Fisher Scientific, #71–1500, 1:100), anti-Claudin-4 (Abcam, #ab53156, 1:200), anti-phospho-JNK (Affinity, #AF3318, 1:200), anti-PDE4D (Abcam, #ab171750, 1:200), anti-phospho-SGK1 (#36–002, 1:200, Millipore), at 4 °C overnight. Then, cells or lung sections were incubated with Donkey anti-Mouse IgG (H + L), Alexa Fluor^TM^ 488 (Thermo Fisher Scientific, #A-21206, 1:500) or Donkey anti-Rabbit IgG (H + L), Alexa Fluor™ 568 (Thermo Fisher Scientific, #A-10042, 1:500) for 1 hr at room temperature. Finally, the cell nuclei were labeled with 4’,6-diamidino-2-phenylindole (DAPI, D9542, Sigma, USA) and visualized using confocal microscopy (TCS-SP5, Leica, Germany).

### SARS-CoV-2 infection in mice

Adenovirus 5-human ACE2 (Ad5-hACE2) transgenic BALB/c mice 6–8 weeks old were infected with 1×10^5^ plaque-forming units (PFU) of SARS-CoV-2, as previously described.^65^ The mice were sacrificed at day 4 post infection, and the lung samples were fixed in formalin and embedded in paraffin for further immunofluorescence assay. All work with SARS-CoV-2 was strictly confined in the Biosafety Level 3 Laboratory of Guangzhou Customs Technology Center (Guangzhou, China). All experimental procedures were approved by the Institutional Animal Care and Use Committees of the Guangzhou Medical University (Guangzhou, China).

### Mouse model of SARS-CoV-2 E protein stimulation

Male ICR mice, weighing 25–30 g, were obtained from the Laboratory Animal Center of Sun Yat-sen University (Guangzhou, China). Mice were anesthetized with tribromoethanol [0.2 ml/10 g body weight of a 1.25% (vol/vol) solution]. For the E protein-treated groups, different concentrations of SARS-CoV-2 E protein was administered by intratracheal instillation dissolved in 50 μl saline, and mice in the control group were instilled with an equal volume of saline. EMD638683 (10 mg/kg) or rolipram (10 mg/kg) was injected intraperitoneally 1 hr before E protein stimulation. After 24 h, all mice were sacrificed by CO_2_ asphyxiation, and lung tissue samples were collected for subsequent experiments. All procedures were performed in strict accordance with the animal use protocols approved by the Sun Yat-sen University Institutional Animal Care and Use Committee (Guangzhou, China).

### Lung histopathology

The lungs were fixed in 4% paraformaldehyde and embedded in paraffin. Paraffin sections (4 μm) of lungs were stained with hematoxylin-eosin (HE) to visualize histopathological changes and to assess the extent of inflammation induced by E protein. HE staining images were obtained with an Optical Microscope (Nikon, Tokyo, Japan).

### Glutathione S-transferase (GST) pull-down assay

SARS-CoV-2 E protein cDNA was cloned in-frame with GST in the prokaryotic expression vector pGEX-4T-1, and the fusion protein was expressed in *Escherichia coli*. GST fusion proteins were subsequently purified via affinity chromatography with glutathione-Sepharose and immobilized on GST resin. GST protein (negative control) and GST-tagged SARS-CoV-2 E protein were used to pull down His-tagged TLR2 or TLR4 recombinant protein (Zoonbio Biotechnology, China). After washing with PBS, the bound proteins were eluted with elution buffer and protein interactions were verified by SDS-polyacrylamide gel electrophoresis (SDS-PAGE) and western blotting analysis.

### Ussing chamber measurements

Electrophysiological analyses of airway epithelial cells were performed by using short circuit current (*I*_SC_) technique, as described previously.^11^ Briefly, 16HBE14o- cells were grown on permeable supports (Millipore, USA) and mounted in an Ussing chamber. The transepithelial *I*_SC_ through 16HBE14o- cell monolayers was measured with an epithelial voltage clamp (MODEL VCC MC6, Physiologic Instruments, USA). Forskolin was added apically to activate CFTR as previously described.^66^

### Isolation and culture of primary mouse airway epithelial cells

Mouse airway epithelial cells were cultured as described previously.^67^ Briefly, male ICR mice were euthanized using CO_2_ asphyxia and the trachea was isolated and immersed into Hanks’ balanced salt solution containing 1% (v/v) penicillin−streptomycin (Hyclone, USA). Subsequently, the tissues were digested with 0.25% (w/v) trypsin (Gibco, USA) overnight at 4 °C. The digested trachea was removed and the epithelial cells were collected by centrifugation at 400 *g* for 4 min. Cells were cultured in DMEM/Nutrient Mixture F-12 (Hyclone, USA) medium supplemented with 3% (v/v) fetal bovine serum (Gibco, USA) and 1% (v/v) penicillin−streptomycin (Hyclone, USA) at 37 °C in an atmosphere of 5% CO_2_. On day 3, the cells were harvested for subsequent experiments.

### Intracellular Cl^−^ measurement

Airway epithelial cells were seeded on glass cover-slips, and the concentration of intracellular Cl^−^ was monitored through recording the changes of fluorescence intensity of N-(ethoxycarbonylmethyl)−6-methoxyquinolinium bromide (MQAE), a chloride-sensitive indicator dye as described previously.^35,64^

### cAMP enzyme-linked immunosorbent assay

The content of intracellular cAMP was measured by performing enzyme-linked immunosorbent assay (ELISA) with the cAMP Parameter Assay Kit (R&D Systems, USA), according to the manufacturer’s protocol.

### Isolation and culture of primary human airway epithelial cells (hPAECs)

The hPAECs were isolated from the bronchial brushing specimens obtained from the outpatient clinics of the First Affiliated Hospital of Guangzhou Medical University (Guangzhou, China). All study participants provided written informed consent, and this study was approved by the Ethics Committee of The First Affiliated Hospital of Guangzhou Medical University. Upon collection, the airway epithelial cells were detached from the brush and subsequently centrifuged at 500 g for 5 min. The cells were then re-suspended in PneumaCult™-Ex Plus Medium (STEMCELL) containing 1% (v/v) penicillin and streptomycin, and cultured at 37 °C in a humidified atmosphere of 5% CO_2_.

### Statistical analysis

Data are presented as the mean ± standard deviation (SD). Differences between two groups were assessed with Student’s two-tailed *t*-test, and for multiple comparisons, one-way ANOVA followed by Bonferroni was used. Statistical analyses were conducted using Origin Pro software (OriginLab Corporation, MA, USA). *P* < 0.05 was considered statistically significant for the comparisons.

### Supplementary information

SARS-CoV-2 envelope protein impairs airway epithelial barrier function and exacerbates airway inflammation via increased intracellular Cl^−^ concentration

## Data Availability

All data supporting the findings of this research are available within the article and its supplementary information or from the corresponding author upon reasonable request.
