# Supplementary material for: SARS-CoV-2 envelope protein impairs airway epithelial barrier function and exacerbates airway inflammation via increased intracellular Cl− concentration
Source: Signal Transduct Target Ther. 2024 Mar 25;9:74. doi: 10.1038/s41392-024-01753-z (PMC10963779; doi:10.1038/s41392-024-01753-z)
Supplement: Supplementary file 1 — SARS-CoV-2 envelope protein impairs airway epithelial barrier function and exacerbates airway inflammation via increased intracellular Cl− concentration [file 41392_2024_1753_MOESM1_ESM.docx]

Supplementary Materials for

**SARS-CoV-2 envelope protein impairs airway epithelial barrier function and exacerbates airway inflammation via increased intracellular Cl^−^ concentration**

Jian-Bang Xu†^1^, Wei-Jie Guan†^1,2,3,*^, Yi-Lin Zhang†^4^, Zhuo-Er Qiu^4^, Lei Chen^4^, Xiao-Chun Hou^4^, Junqing Yue^1,3^, Yu-Yun Zhou^4^, Jie Sheng^4^, Lei Zhao^1,5^, Yun-Xin Zhu^4^, Jing Sun^1,3^, Jincun Zhao^1,3^, Wen-Liang Zhou^4,*^, Nan-Shan Zhong^1,3,*^

Correspondence should be addressed to nanshan@vip.163.com; lsszwl@mail.sysu.edu.cn; battery203@163.com.

†Jian-Bang Xu, Wei-Jie Guan and Yi-Lin Zhang contributed equally to this work and are co-first authors.

**This file includes:**

Supplementary Materials and methods

Figures. S1 to S14

Table. S1

**Supplementary Materials and methods**

Cell Counting Kit-8 (CCK-8) assay

16HBE14o- cells were seeded in 96-well plates and cultured to 80% confluence, followed by incubation with or without SARS-CoV-2 E protein for 2 hrs. The cell viability of 16HBE14o- cells was then assayed using Enhanced CCK-8 (Meilunbio, China), according to manufacturer's instruction. Finally, the absorbance at 450 nm was detected using a microplate reader (BioTek, USA).

Plasmid transfection

BEAS-2B cells were inoculated in the 24-well cell culture plate and were transfected with plasmid pCMV3-2019-nCoV-ENV (#VG40609-UT, Sino Biological, China) or plasmid pCMV/hygro-Negative Control Vector (#CV001, Sino Biological, China) using Lipo3000 transfection reagent (#L3000001, Thermo Fisher Scientific, USA), following the manufacture's protocol.


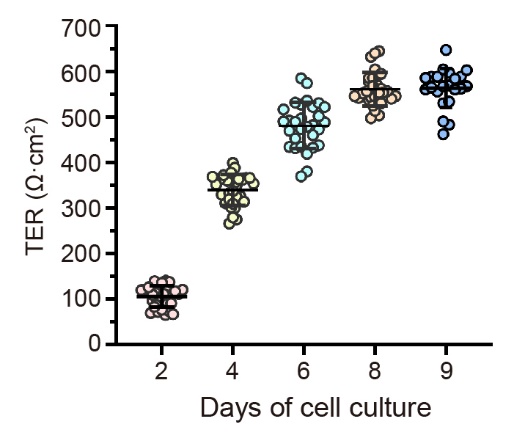


**Supplementary** **Fig. S1** The development of epithelial confluent monolayer of airway epithelial cells. Transepithelial electrical resistances (TER) values were measured in 16HBE14o- cells at various time points (*n* = 22-30). Data are shown as means ± S.D.


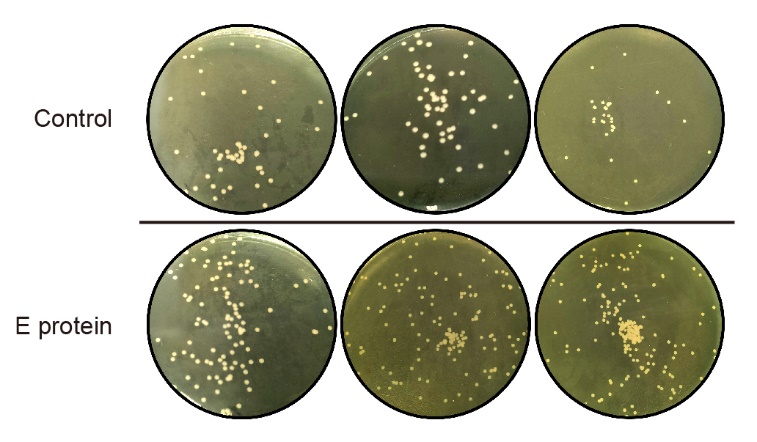


**Supplementary** **Fig. S2** Effect of SARS-CoV-2 envelope (E) protein on the penetration of *Pseudomonas aeruginosa* strain PAO1 through the monolayer of airway epithelial cells. Agar plates showing colony forming unit of PAO1 penetrated into the basolateral medium in the absence or presence of E protein (50 μg/ml) stimulation in 16HBE14o- cells.


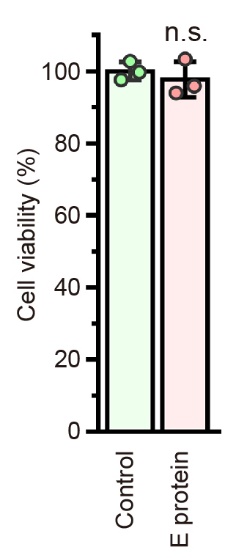


**Supplementary** **Fig. S3** Effect of SARS-CoV-2 envelope (E) protein on cell viability of airway epithelial cells. The cell viability of 16HBE14o- cells was evaluated with or without E protein (50 μg/ml) stimulation for 2 hrs. Data are shown as means ± S.D. (*n* = 3, ns = not significant).


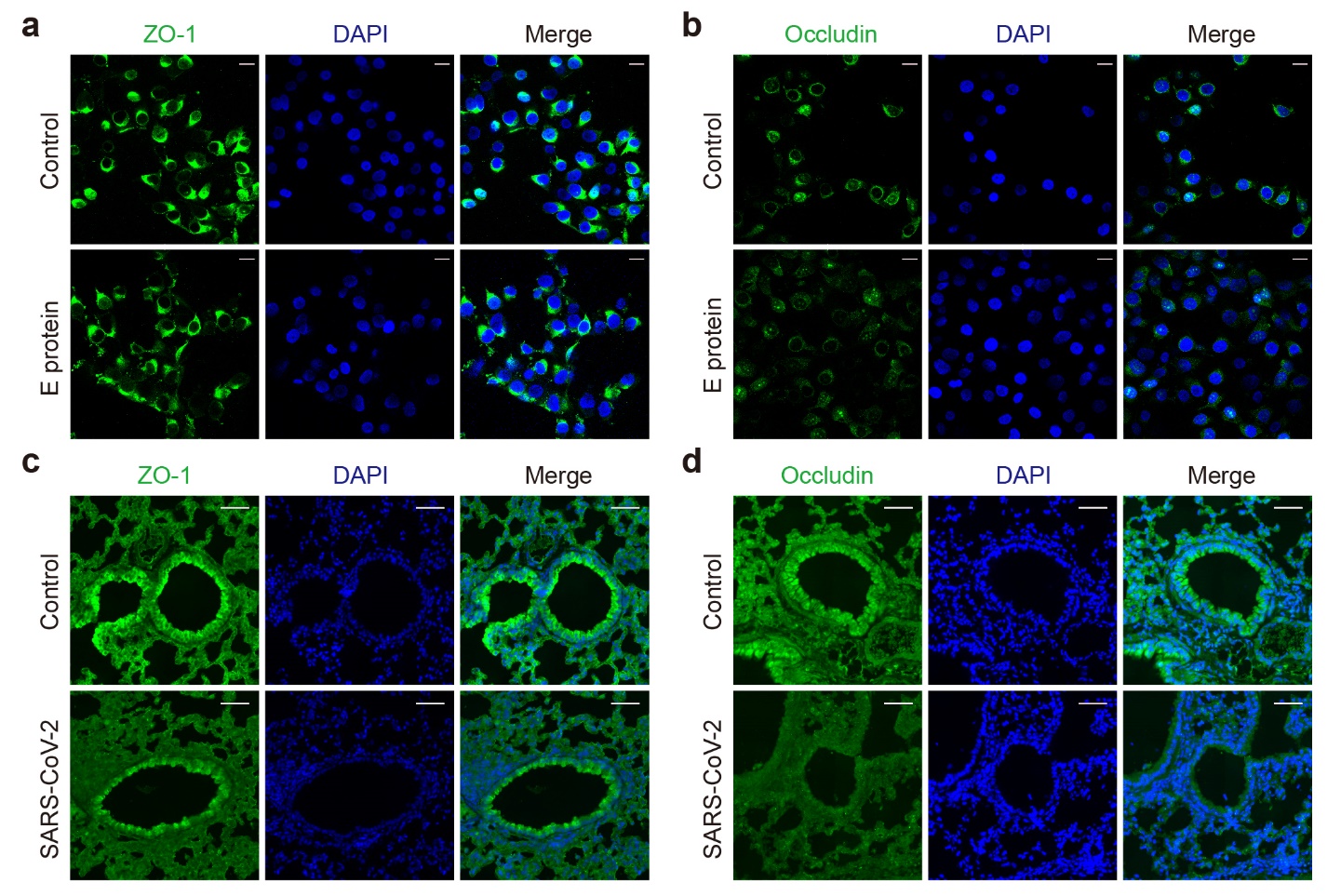


**Supplementary** **Fig. S4** Effect of SARS-CoV-2 envelope (E) protein on the expression of tight junction–associated proteins. **a-b** Immunofluorescence images showing the expression of ZO-1 and occludin, in the absence or presence of E protein (50 μg/ml) stimulation in BEAS-2B cells. Scale bar = 20 μm. **c-d** Immunofluorescence staining of lung slices showing the expression of ZO-1 and occludin in Ad5-hACE2 transgenic mice with or without SARS-CoV-2 infection (1 × 10^5^ PFU). Scale bar = 50 μm.


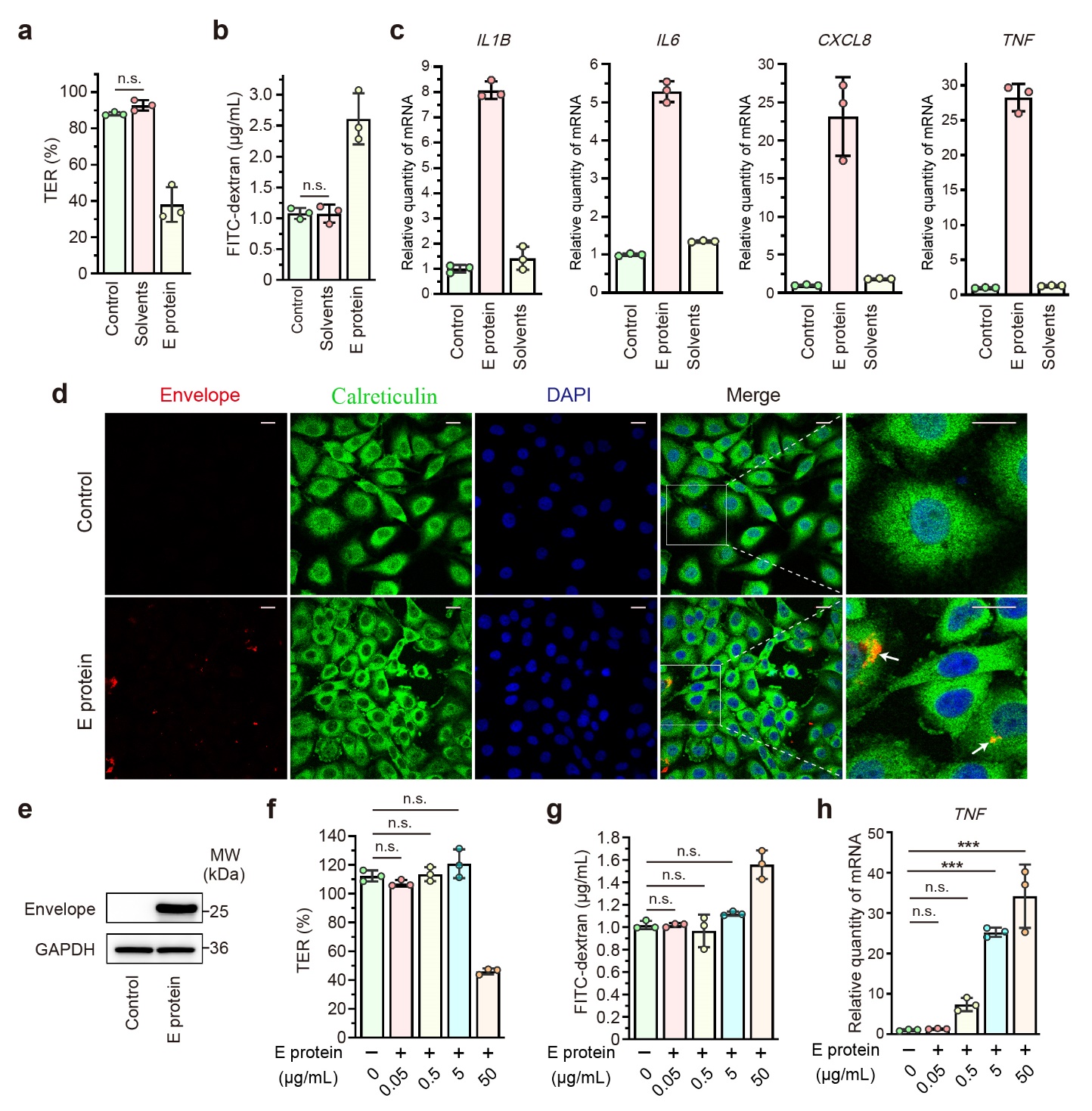


**Supplementary** **Fig. S5** The effect of the different concentrations of E protein and its solvent on the barrier function and the expression of pro-inflammatory cytokines, and the presence of SARS-CoV-2 envelope (E) protein in airway epithelial cells. **a-b** 16HBE14o- monolayers were stimulated with E protein (50 μg/ml) or the solvents apically for 3 hrs. **a** TER values were measured and shown relative to the TER before the solvents or E protein stimulation (n = 3). **b** FD4 flux across cell monolayers in the presence of the solvents or E protein (*n* = 3). **c** Quantitative real-time PCR analyses showing the effects of the E protein or the solvents on the expression of pro-inflammatory cytokines/chemokines in BEAS-2B cells stimulated for 12 hrs (*n* = 3). **d** Confocal images showing the immunofluorescence labeling of E protein and calreticulin after E protein stimulation for 6 hrs in BEAS-2B cells. Scale bar = 20 μm. **e** Representative immunoblots showing the expression of E protein in BEAS-2B cells after stimulation for 12 hrs. GAPDH served as a loading control. **f-g** 16HBE14o- monolayers were stimulated with E protein (50 μg/ml) apically for 3 hrs. **f** TER values were measured and shown relative to the TER before the stimulation of different concentrations of E protein (*n* = 3). **g** FD4 flux across cell monolayers in the presence of the different concentrations of E protein (*n* = 3). **h** Quantitative real-time PCR analyses showing the effects of the different concentrations of E protein on the expression of TNF-α in BEAS-2B cells stimulated for 12 hrs (*n* = 3).


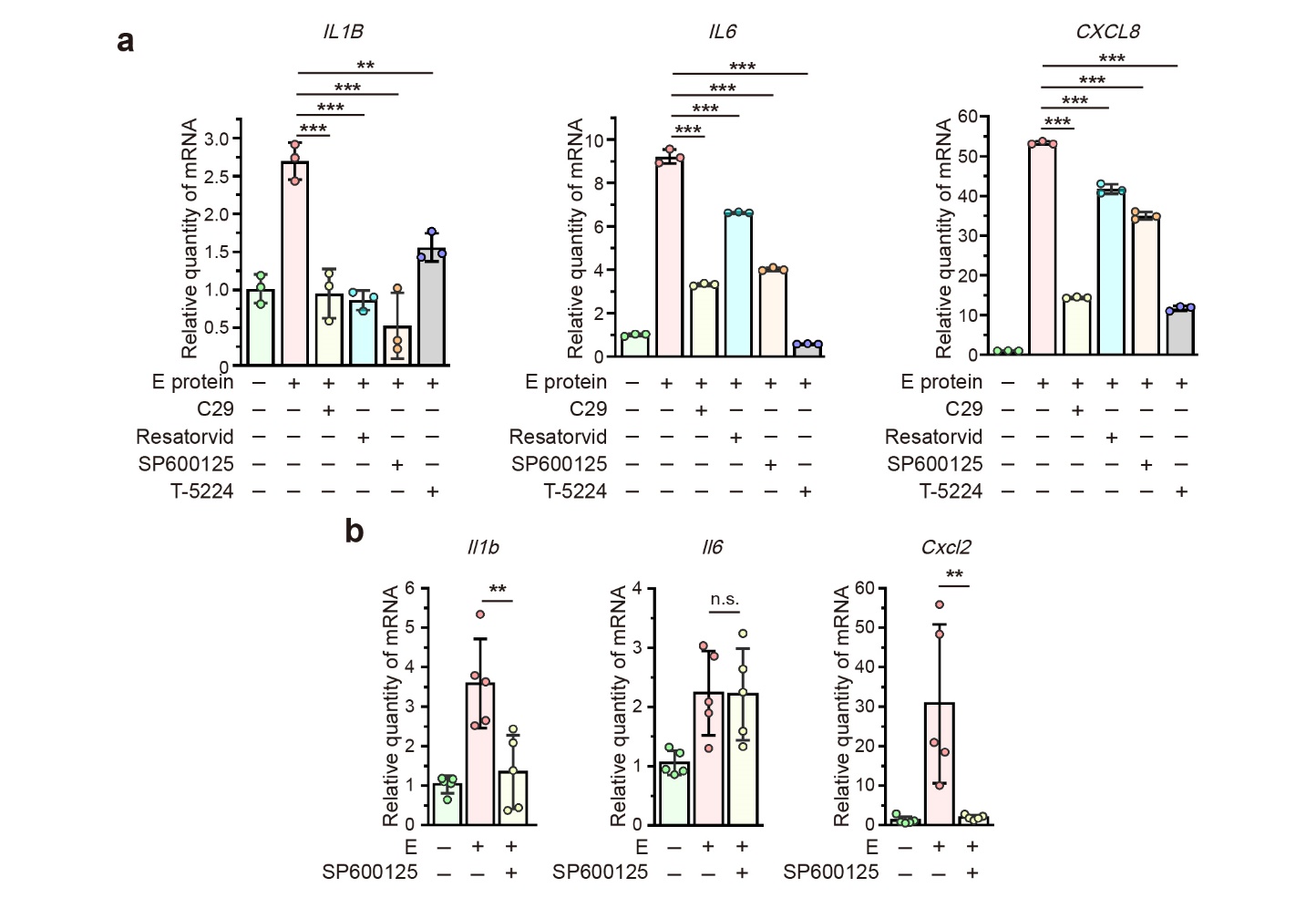


**Supplementary** **Fig. S6** Effects of inhibition of TLR2, TLR4, JNK or AP-1 on airway epithelial inflammation triggered by SARS-CoV-2 envelope (E) protein. **a** Quantitative real-time PCR analyses showing the effects of the TLR2 inhibitor C29 (50 μM), the TLR4 inhibitor Resatorvid (5 μM), the JNK inhibitor SP600125 (10 μM) and the AP-1 inhibitor T-5224 (10 μM) on the expression of pro-inflammatory cytokines/chemokines in BEAS-2B cells stimulated with E protein (50 μg/ml) for 12 hrs (*n* = 3). **b** Quantitative real-time PCR analyses showing the effect of SP600125 (30 mg/kg) on the expression of pro-inflammatory cytokines/chemokines in the lung samples from mice after intratracheal instillation of E protein (100 μg/mL) for 24 hrs (*n* = 5). Data are shown as means ± S.D. ns = no significant, ^**^ *P* < 0.01, ^***^ *P* < 0.001 indicated by lines.


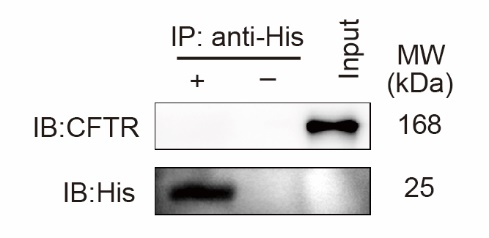


**Supplementary** **Fig. S7** SARS-CoV-2 envelope (E) protein did not interact with cystic fibrosis transmembrane conductance regulator (CFTR) in airway epithelial cells. Co-immunoprecipitates and the total lysates (Input) were analyzed by immunoblotting using anti-CFTR antibody, after incubation with of E protein.


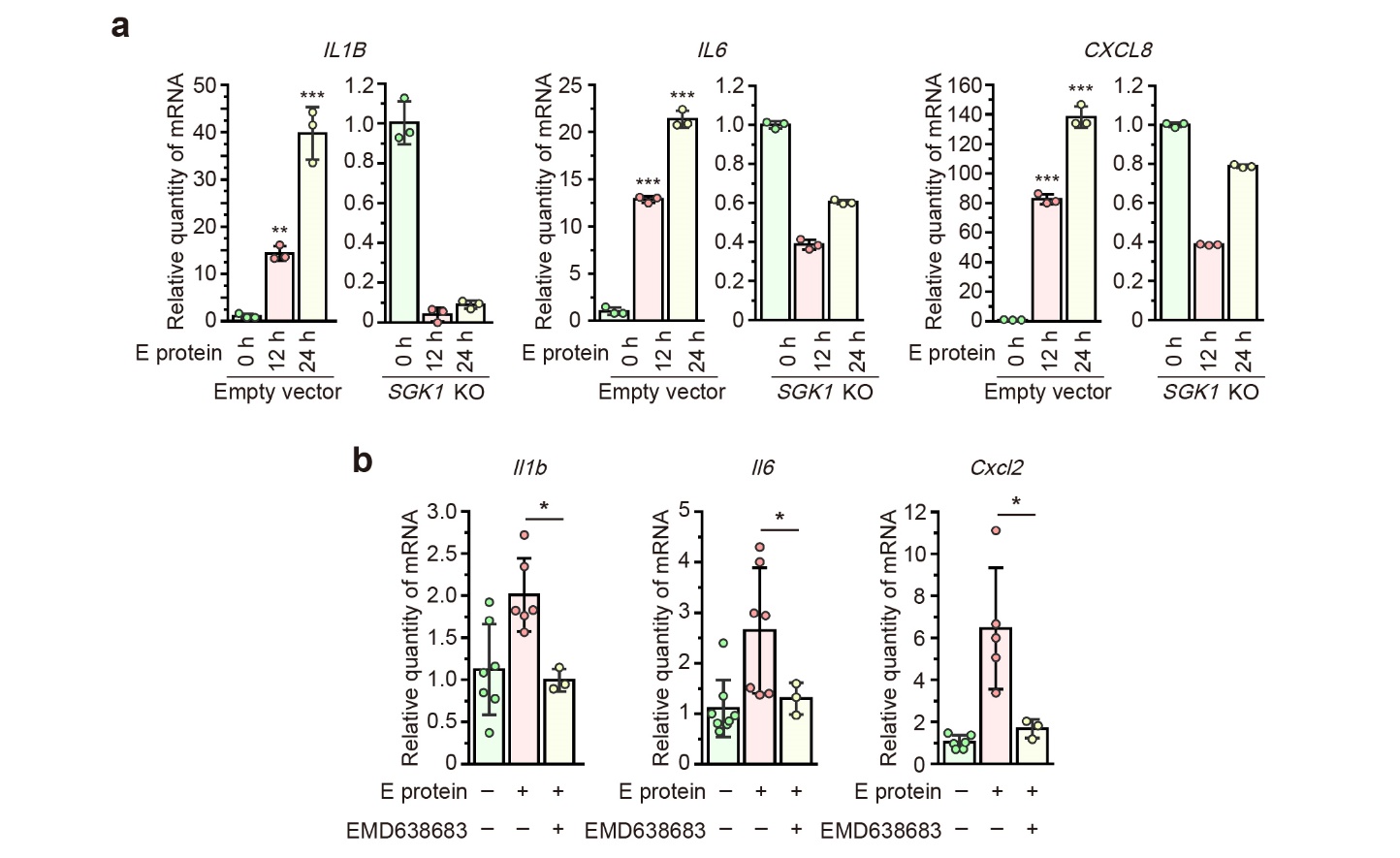


**Supplementary** **Fig. S8** Effects of inhibition or knockout (KO) of serum/glucocorticoid regulated kinase 1 (SGK1) on airway epithelial inflammation triggered by SARS-CoV-2 envelope (E) protein. **a** Quantitative real-time PCR analyses showing the effect of *SGK1* KO on the expression of pro-inflammatory cytokines/chemokines in BEAS-2B cells stimulated with E protein (50 μg/ml) for the indicated time points (*n* = 3). **b** Quantitative real-time PCR analyses showing the effect of EMD638683 (10 mg/kg), the selective inhibitor of SGK1, on the expression of pro-inflammatory cytokines/chemokines in the lung samples from mice after intratracheal instillation of E protein (100 μg/ml) for 24 hrs (*n* = 3-8). Data are shown as means ± S.D. ^*^ *P* < 0.05, ^**^ *P* < 0.01, ^***^ *P* < 0.001 versus the control group or indicated by lines.


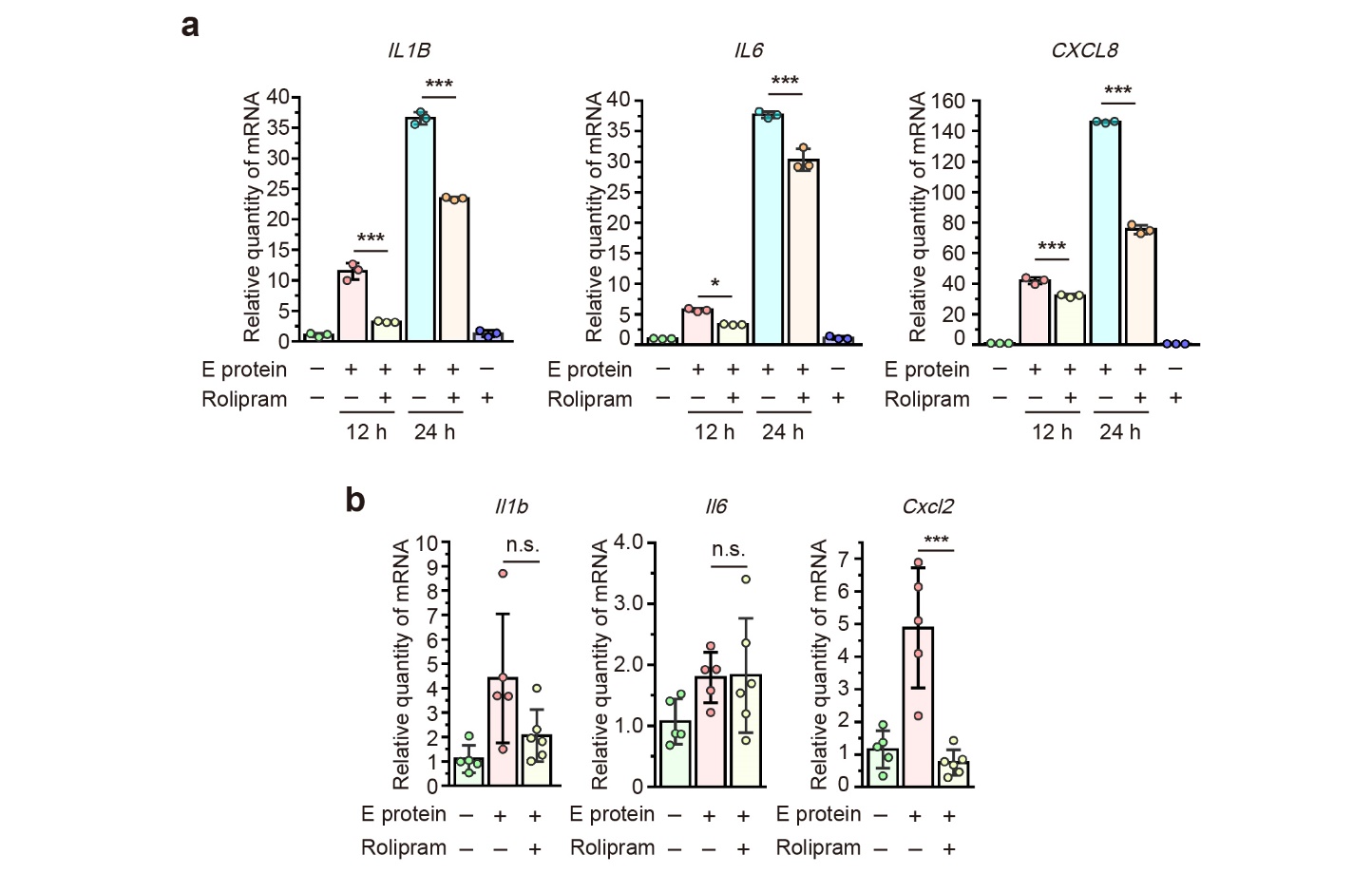


**Supplementary** **Fig. S9** Effects of inhibition of Phosphodiesterase 4 (PDE4) on airway epithelial inflammation triggered by SARS-CoV-2 envelope (E) protein. **a** Quantitative real-time PCR analyses showing the effect of rolipram (20 μM), a selective PDE4 inhibitor, on the expression of pro-inflammatory cytokines/chemokines in BEAS-2B cells stimulated with E protein (50 μg/ml) for the indicated different time points (*n* = 3). **b** Quantitative real-time PCR analyses showing the effect of rolipram (10 mg/kg) on the expression of pro-inflammatory cytokines/chemokines in the lung samples derived from mice after intratracheal instillation of E protein (100 μg/ml) for 24 hrs (n = 5-6). Data are shown as means ± S.D. ns = not significant, ^*^ *P* < 0.05, ^***^ *P* < 0.001 indicated by lines.


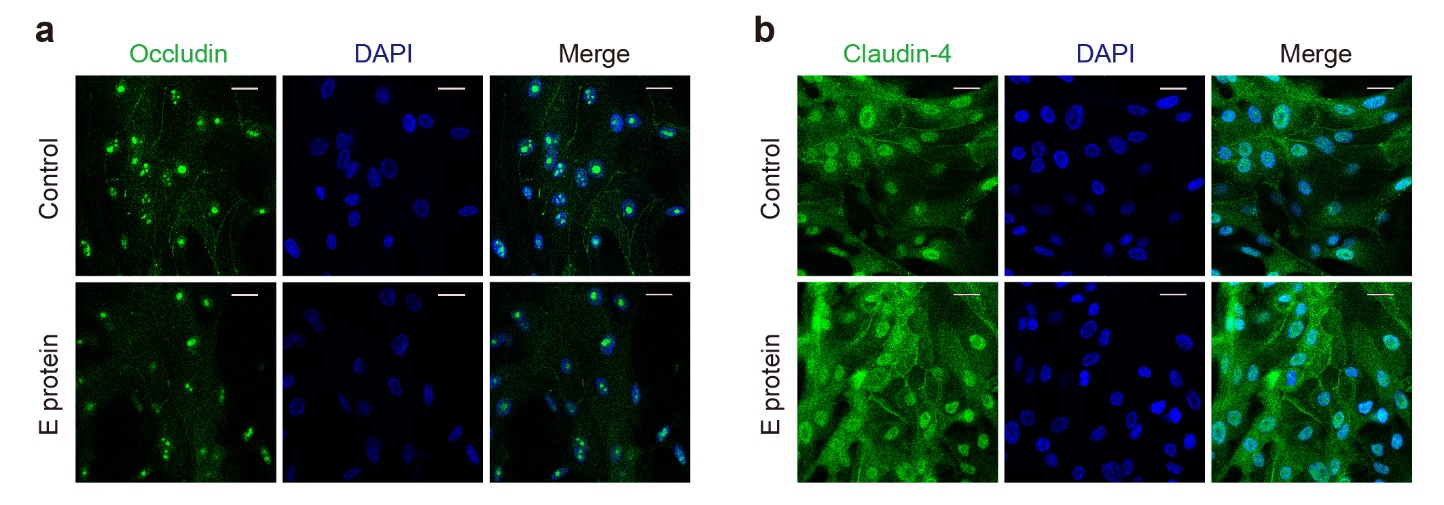
 **Supplementary** **Fig. S10** Effect of SARS-CoV-2 envelope (E) protein on the expression of tight junction–associated proteins in human primary cultured airway epithelial cells (hPAECs). Immunofluorescence images showing the expression of occludin and claudin-4 in hPAECs, in the absence or presence of E protein (50 μg/ml) stimulation.


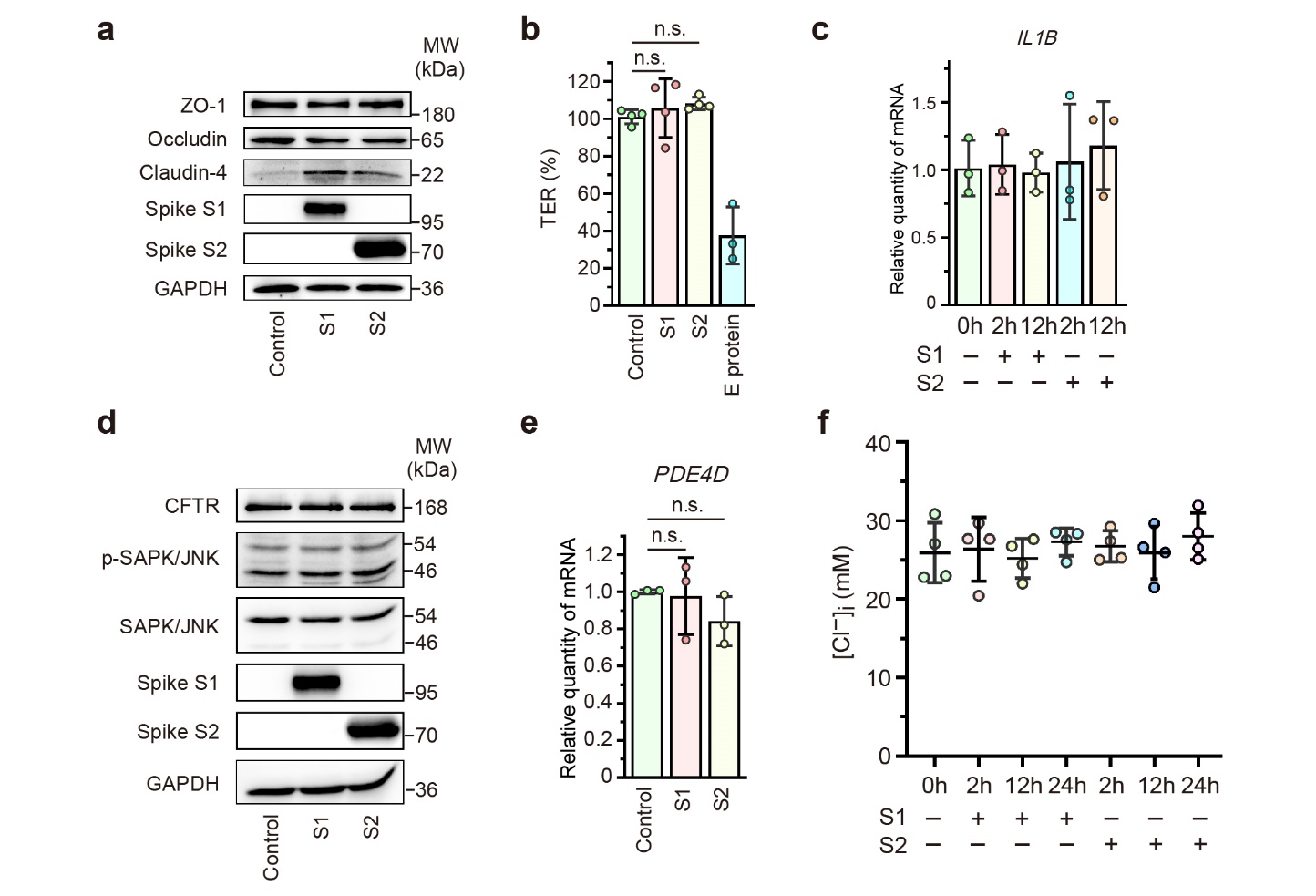


**Supplementary** **Fig. S11** Effects of SARS-CoV-2 Spike (S) protein on airway epithelial barrier and intracellular Cl^−^ signaling. **a** Representative immunoblots showing the expression level of ZO-1, occludin and claudin-4 in BEAS-2B cells after stimulation with S protein (50 μg/ml) for 12 hrs. GAPDH served as a loading control. **b** Transepithelial resistance (TER) values were measured and shown relative to the TER before S protein or E protein stimulation (*n* = 3-4). **c** Quantitative real-time PCR analyses showing the effect of S protein on the expression of IL-1β in BEAS-2B cells stimulated with S protein (50 μg/ml) for 12hrs (*n* = 3). **d** Representative immunoblots showing the expression of cystic fibrosis transmembrane conductance regulator (CFTR) and the phosphorylation level of JNK in BEAS-2B cells after stimulation with the S protein (50 μg/ml) for 12 hrs. GAPDH served as a loading control. **e** Quantitative real-time PCR analyses showing the effect of S protein on the expression of PDE4D in BEAS-2B cells stimulated with S protein (50 μg/ml) for 12hrs (*n* = 3). **f** Intracellular Cl^−^ concentration (Cl^−^]_i_) was measured in BEAS-2B cells stimulated with S protein (50 μg/ml) for the indicated time points (*n* = 4 cells for each group). Data are shown as means ± S.D. ns = not significant.


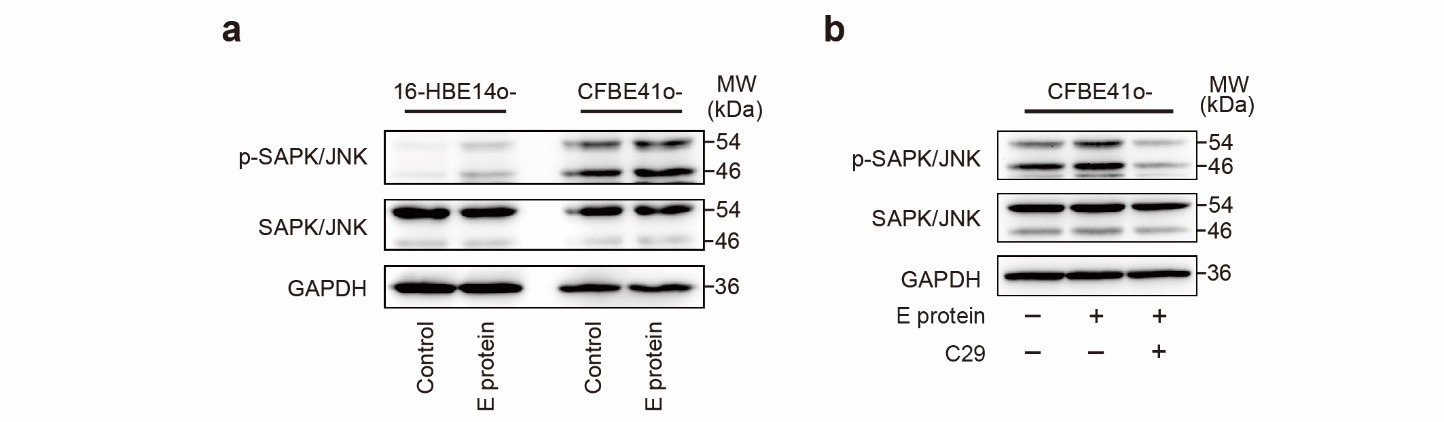


**Supplementary** **Fig. S12** Involvement of TLR in the enhanced phosphorylation of JNK in CFBE41o- cells after stimulation with SARS-CoV-2 envelope (E) protein. **a** Representative immunoblots showing the phosphorylation level of JNK in 16HBE14o- and CFBE41o- cells after E protein (50 μg/ml) stimulation for 12 hrs. GAPDH served as a loading control. **b** Representative immunoblots showing the effect of C29 (50 μM), an inhibitor of TLR2, on the phosphorylation level of JNK in CFBE41o- cells after stimulation with E protein (50 μg/ml) for 12 hrs. GAPDH served as a loading control.


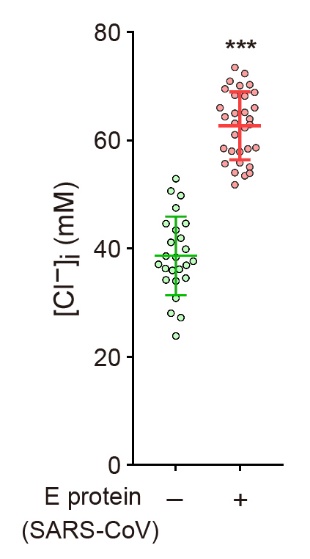


**Supplementary** **Fig. S13** Effect of SARS-CoV envelope (E) protein on the intracellular Cl^−^ concentration ([Cl^−^]_i_) of human primary cultured airway epithelial cells (hPAECs). [Cl^−^]_i_ was measured in hPAECs stimulated with SARS-CoV E protein (50 μg/ml) for 12 hrs (*n* = 25-31 cells for each group). Data are shown as means ± S.D. ^***^ *P* < 0.001 versus the control group.


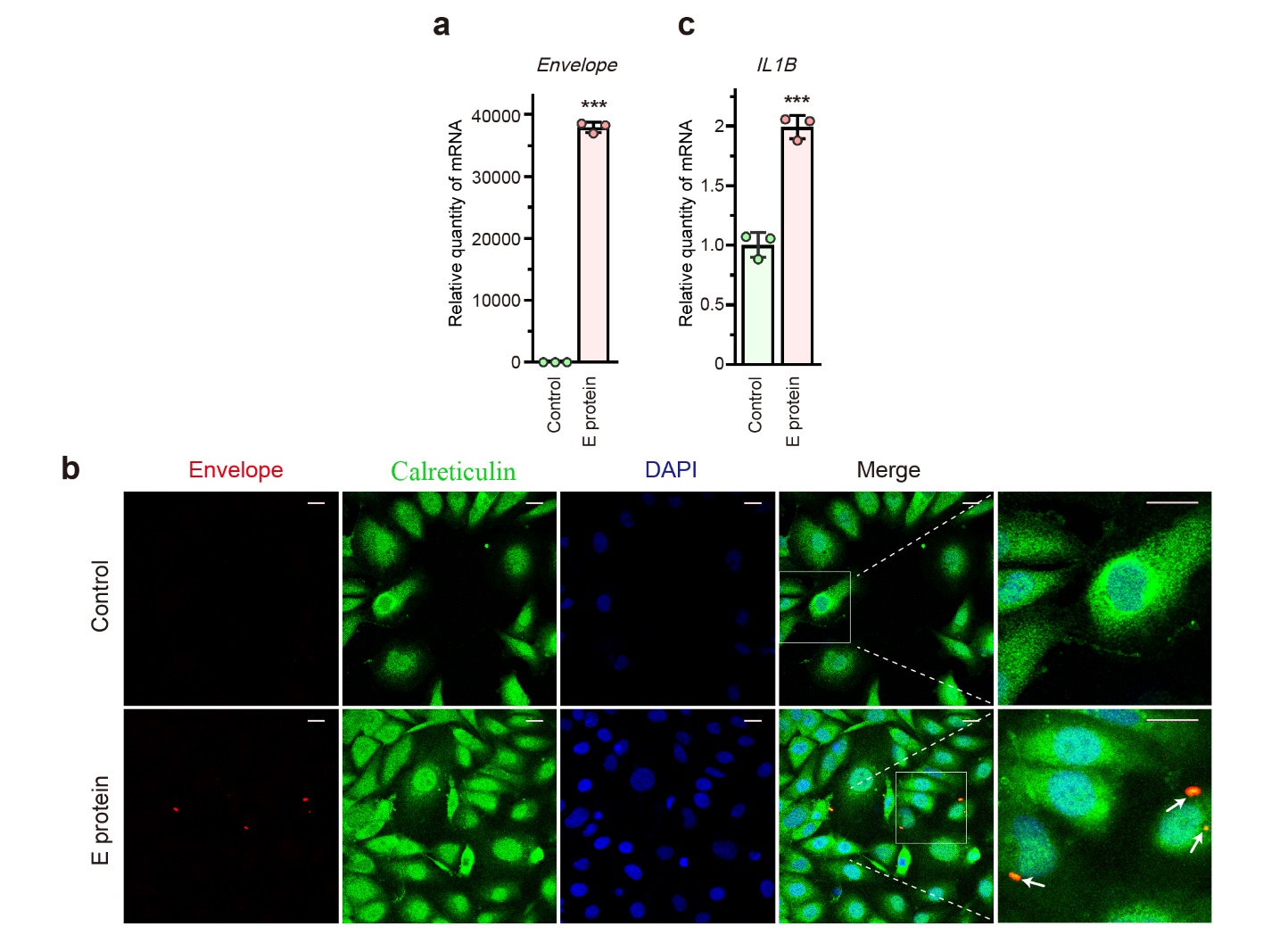


**Supplementary** **Fig. S14** Endogenous expression of SARS-CoV-2 envelope (E) protein in airway epithelial cells and its effects on the expression of pro-inflammatory cytokine. **a** Quantitative real-time PCR analyses showing the expression of E gene after transfection of the envelope protein Gene ORF cDNA clone expression plasmid for 24 hrs (*n* = 3). **b** Confocal images showing the immunofluorescence labeling of E protein and calreticulin after E protein plasmid transfection for 36 hrs in BEAS-2B cells. Scale bar = 20 μm. **c** Quantitative real-time PCR analyses showing the expression of IL-1β after transfection of the envelope protein Gene ORF cDNA clone expression plasmid for 24 hrs (*n* = 3).

**Table S1** Quantitative real-time PCR primers used in this study

| Primer name | Sequence |
| --- | --- |
| *TJP1* Forward Primer  *TJP1* Reverse Primer  *OCLN* Forward Primer  *OCLN* Reverse Primer  *CLDN4* Forward Primer  *CLDN4* Reverse Primer  *IL1B* Forward Primer  *IL1B* Reverse Primer  *IL6* Forward Primer  *IL6* Reverse Primer  *CXCL8* Forward Primer  *CXCL8* Reverse Primer  *TNF* Forward Primer  *TNF* Reverse Primer  *GAPDH* Forward Primer  *GAPDH* Reverse Primer  *PDE4A* Forward Primer  *PDE4A* Reverse Primer  *PDE4B* Forward Primer  *PDE4B* Reverse Primer  *PDE4C* Forward Primer  *PDE4C* Reverse Primer  *PDE4D* Forward Primer  *PDE4D* Reverse Primer  *Il1b* Forward Primer  *Il1b* Reverse Primer  *Il6* Forward Primer  *Il6* Reverse Primer  *Cxcl2* Forward Primer  *Cxcl2* Reverse Primer  *Tnf* Forward Primer  *Tnf* Reverse Primer  *Pde4d* Forward Primer  *Pde4d* Reverse Primer  *Hprt* Forward Primer  *Hprt* Reverse Primer  *Envelope* Forward Primer  *Envelope* Reverse Primer | ACCAGTAAGTCGTCCTGATCC  TCGGCCAAATCTTCTCACTCC  ACAAGCGGTTTTATCCAGAGTC  GTCATCCACAGGCGAAGTTAAT  GGGGCAAGTGTACCAACTG  GACACCGGCACTATCACCA  TTCGACACATGGGATAACGAGG  TTTTTGCTGTGAGTCCCGGAG  CCTGAACCTTCCAAAGATGGC  TTCACCAGGCAAGTCTCCTCA  ACTGAGAGTGATTGAGAGTGGAC  AACCCTCTGCACCCAGTTTTC  GAGGCCAAGCCCTGGTATG  CGGGCCGATTGATCTCAGC  TGCACCACCAACTGCTTAGC  GGATGCAGGGATGATGTTCT  GGGGTGAAGACCGATCAAGAA  CGACACGCAAAAGATGTTCAG  CGGCTGGGAGAGAGGGTTATT  TCTCCAGAGGTCGATCCCAAG  GAGACGCTAGACGAGCTGGA  GTGGGTCAACTCCCGGTTC  TGTGTGACAAGCACAATGCTTCC  CACGATTGTCCTCCAAAGTGTCC  AACCTGCTGGTGTGTGACGTTC  CAGCACGAGGCTTTTTTGTTGT  AGTTGCCTTCTTGGGACTGATG  CAGGTCTGTTGGGAGTGGTATC  CCTGCCAAGGGTTGACTTCA TTCTGTCTGGGCGCAGTG  GACGTGGAAGTGGCAGAAGAG  TGCCACAAGCAGGAATGAGA  TTTTGCCAGTGCAATACATGATG  CAGAGCGAGTTCCGAGTTTGT  TCAGTCAACGGGGGACATAAA  GGGGCTGTACTGCTTAACCAG  GTTTCGGAAGAGACAGGTACG  AAGCGCAGTAAGGATGGCTA |
